# Lexical Processing Strongly Affects Reading Times But Not Skipping During Natural Reading

**DOI:** 10.1162/opmi_a_00099

**Published:** 2023-10-01

**Authors:** Micha Heilbron, Jorie van Haren, Peter Hagoort, Floris P. de Lange

**Affiliations:** Donders Institute for Brain, Cognition and Behaviour, Radboud University, Nijmegen, The Netherlands; Max Planck Institute for Psycholinguistics, Nijmegen, The Netherlands; University of Amsterdam, Amsterdam, The Netherlands

**Keywords:** reading, eye movements, prediction, preview, Bayesian reader, neural networks

## Abstract

In a typical text, readers look much longer at some words than at others, even skipping many altogether. Historically, researchers explained this variation via low-level visual or oculomotor factors, but today it is primarily explained via factors determining a word’s lexical processing ease, such as how well word identity can be predicted from context or discerned from parafoveal preview. While the existence of these effects is well established in controlled experiments, the relative importance of prediction, preview and low-level factors in natural reading remains unclear. Here, we address this question in three large naturalistic reading corpora (*n* = 104, 1.5 million words), using deep neural networks and Bayesian ideal observers to model linguistic prediction and parafoveal preview from moment to moment in natural reading. Strikingly, neither prediction nor preview was important for explaining word skipping—the vast majority of explained variation was explained by a simple oculomotor model, using just fixation position and word length. For reading times, by contrast, we found strong but independent contributions of prediction and preview, with effect sizes matching those from controlled experiments. Together, these results challenge dominant models of eye movements in reading, and instead support alternative models that describe skipping (but not reading times) as largely autonomous from word identification, and mostly determined by low-level oculomotor information.

## INTRODUCTION

When reading a text, readers move their eyes across the page to bring new information to the centre of the visual field, where perceptual sensitivity is highest. While it may subjectively feel as if the eyes smoothly slide along the text, they in fact traverse the words with rapid jerky movements called *saccades*, followed by brief stationary periods called *fixations*. Across a text, saccades and fixations are highly variable and seemingly erratic: Some fixations last less than 100 ms, others more than 400; and while some words are fixated multiple times, many other words are skipped altogether (Dearborn, 1906; Rayner & Pollatsek, 1987). What explains this striking variation?

Historically, researchers have pointed to low-level non-linguistic factors like word length, oculomotor noise, or the relative position where the eyes happen to land (Bouma & de Voogd, 1974; Buswell, 1920; Dearborn, 1906; O’Regan, 1980). Such explanations were motivated by the idea that oculomotor control was largely *autonomous*. In this view, readers can adjust saccade lengths and fixation durations to global characteristics like text difficulty or reading strategy, but not to subtle word-by-word differences in language processing (Bouma & de Voogd, 1974; Buswell, 1920; Dearborn, 1906; Morton, 1964).

As reading was studied in more detail, however, it became clear that the link between eye movements and cognition was more direct. For instance, it was found that fixation durations were shorter for words with higher frequency (Inhoff, 1984; Rayner, 1977). Eye movements were even shown to depend on how well a word’s identity could be inferred *before* fixation. Specifically, researchers found that words are read faster and skipped more often if they are *predictable* from linguistic context (Balota et al., 1985; Ehrlich & Rayner, 1981) or if they are identifiable from a *parafoveal preview* (McConkie & Rayner, 1975; Rayner, 1975; Schotter et al., 2012).

These demonstrations of a direct link between eye movements and language processing overturned the autonomous view, replacing it by cognitive accounts describing eye movements during reading as largely, if not entirely, controlled by linguistic processing (Clifton et al., 2016; Reichle et al., 2003). Today, many studies still build on the powerful techniques like gaze-contingent displays that helped overturn the autonomous view, but now ask much more detailed questions, like whether word identification is a distributed or sequential process (Kliegl et al., 2006, 2007); how many words can be processed in the parafovea (Rayner et al., 2007); at which level they are analysed (Hohenstein & Kliegl, 2014; Pan et al., 2021), and how this may differ between writing systems or orthographies (Tiffin-Richards & Schroeder, 2015; Yan et al., 2010).

Here, we ask a different, perhaps more elemental question: how much of the variation in eye movements do linguistic prediction, parafoveal preview, and non-linguistic factors each explain? That is, how important are these factors for determining how the eyes move during reading? Dominant, cognitive models explain eye movement variation primarily as a function of lexical processing. Skipping, for instance, is modelled as the probability that a word is identified before fixation (Engbert & Kliegl, 2003; Engbert et al., 2005; Reichle et al., 2003). Some, however, have questioned this purely cognitive view, suggesting that low-level features like word eccentricity or length might be more important (Brysbaert et al., 2005; Reilly & O’Regan, 1998; Vitu et al., 1995). One particularly relevant analysis comes from Brysbaert et al. (2005). Presenting a meta-analysis on the aggregate effect sizes on word skipping, they argue that the effect of length and distance is so large that skipping may not just be driven by ongoing word identification, but also—and indeed perhaps primarily—by low-level heuristics part of a simple, scanning strategy (Brysbaert et al., 2005). Similarly, one may ask what drives next-word identification: is identifying the next word mostly driven by linguistic predictions (Goodman, 1967) or by parafoveal perception? Remarkably, while it is well-established that both linguistic and oculomotor, and both predictive and parafoveal processing, all affect eye-movements (Brysbaert et al., 2005; Kliegl et al., 2004; Schotter et al., 2012; Staub, 2015), a comprehensive picture of their relative explanatory power is currently missing, perhaps because they are seldom studied all at the same time.

To arrive at such a comprehensive picture we focus on natural reading, analysing three large datasets of participants reading passages, long articles, and even an entire novel—together encompassing 1.5 million (un)fixated words, across 108 individuals (Cop et al., 2017; Kennedy, 2003; Luke & Christianson, 2018). We use a model-based approach: instead of manipulating word predictability or perturbing parafoveal perceptibility, we combine deep neural language modelling (Radford et al., 2019) and Bayesian ideal observer analysis (Duan & Bicknell, 2020) to quantify how much information about next-word identity is conveyed by both prediction and preview, on a moment-by-moment basis. Our model-based analysis is quite different from the experimental approach, especially in the case of parafoveal preview which is generally studied with a boundary paradigm. However, the underlying logic is the same: in the boundary paradigm, eye movements are compared between conditions in which the preview is informative (valid) and when it conveys no (or incorrect) information about word identity. We—following (Bicknell & Levy, 2010; Duan & Bicknell, 2020)—simply replace this categorical contrast with a more continuous analysis, quantifying the subtle word-by-word variation in the *amount* of information conveyed by the prior preview. In this sense, our approach can be seen as an extension and refinement of the seminal analyses by Brysbaert and colleagues, allowing for instance to quantify not just the effect of word length on skipping—but also, simultaneously, estimate and control for the effect word length has on a word’s prior parafoveal identifiability.

In this way, our word-by-word, information-theoretic analysis brings us closer to the underlying mechanisms than analysing effect sizes in the aggregate. However, we want to stress we use these models as *normative* models to estimate how much information is *in principle* available from prediction and preview at each moment, but do not take these as processing models of human cognition (see Methods and Discussion for a more extensive comparison of our model-based approach and traditional methods). Such a broad-coverage model-based approach has been applied to predictability effects on reading before (Frank et al., 2013; Goodkind & Bicknell, 2018; Kliegl et al., 2004; Luke & Christianson, 2016; Shain et al., 2022; Smith & Levy, 2013), but either without considering preview or only through coarse heuristics such as using word frequency as a proxy for parafoveal identifiability (Kennedy et al., 2013; Kliegl et al., 2006; Pynte & Kennedy, 2006) (but see Duan & Bicknell, 2020). By contrast, we explicitly model both, in addition to low-level explanations like autonomous oculomotor control. To assess explanatory power, we use set theory to derive the unique and shared variation in eye movements explained by each model.

To preview the results, this revealed a striking dissociation between skipping and reading times. For word skipping, the overwhelming majority of explained variation could be explained—mostly *uniquely* explained—by a non-linguistic oculomotor model, that explained word skipping just as a function of a word’s distance to the prior fixation position and its length. These two low-level variables explained much more skipping variation than the degree to which a word was identifiable or predictable prior to fixation. For reading times, by contrast, we did find that factors determining a word’s lexical processing explained most variance. In line with dominant models, we found strong effects of both prediction and preview, matching effect sizes from controlled designs. Interestingly, prediction and parafoveal preview seem to operate independently: we found strong evidence against Bayes-optimal integration of the two. Together, these results support and extend the earlier conclusions of Brysbaert and colleagues, while challenging dominant cognitive models of reading, showing that skipping (or the decision of *where* to fixate) and reading times (i.e., *how long* to fixate) are governed by different principles, and that for word skipping, the link between eye movements and cognition is less direct than commonly thought.

## RESULTS

We analysed eye movements from three large datasets of participants reading texts ranging from isolated paragraphs to an entire novel. Specifically, we considered three datasets: Dundee (Kennedy, 2003) (*N* = 10, 51.502 words per participant), Geco (Cop et al., 2017) (*N* = 14, 54.364 words per participant) and Provo (Luke & Christianson, 2018) (*N* = 84, 2.689 words per participant). In each corpus, we analysed both skipping and reading times (indexed by gaze duration), as they are thought to reflect separate processes: the decision of *where* vs. *how long* to fixate, respectively (Brysbaert et al., 2005; Reichle et al., 2003). For more descriptive details about the data across participants and datasets, see Methods and Figures A.5–A.7.

**Figure 1.**
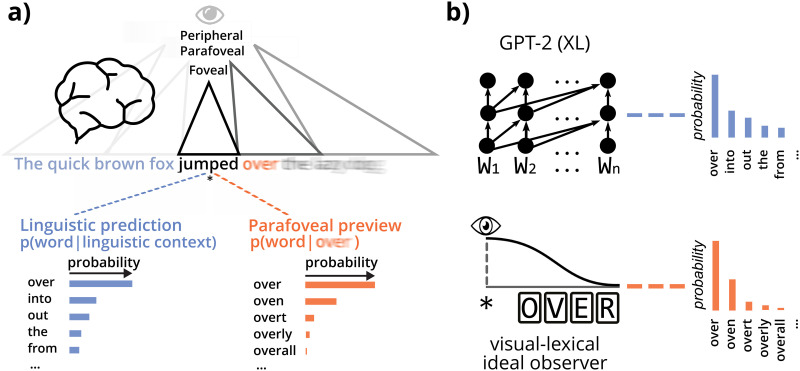
**Quantifying two types of context during natural reading.** (A) Readers can infer the identity of the next word before fixation either by predicting it from context or by discerning it from the parafovea. Both can be cast as a probabilistic inference about the next word, either given the preceding words (prediction, blue) or given a parafoveal percept (preview, orange). (B) To model prediction, we use GPT-2, one of the most powerful publicly available language models (Radford et al., 2019). For preview, we use an ideal observer (Duan & Bicknell, 2020) based on well-established ‘Bayesian Reader’ models (Bicknell & Levy, 2010; Norris, 2006, 2009). Importantly, we do not use either model as a cognitive model *per se*, but rather as a tool to quantify how much information is *in principle* available from prediction or preview on a moment-by-moment basis.

To estimate the effect of linguistic prediction and parafoveal preview, we quantified the amount of information conveyed by both factors for each word in the corpus (for preview, this was tailored to each individual participant, since each word was previewed at a different eccentricity by each participant). To this end, we formalised both processes as a probabilistic belief about the identity of the next word, given either the preceding words (prediction) or a noisy parafoveal percept (preview; see Figure 1A). As such, we could describe these disparate cognitive processes using a common information-theoretic currency. To compute the probability distributions, we used GPT-2 for prediction (Radford et al., 2019) and a Bayesian ideal observer for preview (Duan & Bicknell, 2020) (see Figure 1B and Methods). Note that we use both computational models as *normative* models; tools to estimate how much information is *in principle* available from linguistic context (prediction) or parafoveal perception (preview) on a moment-by-moment basis. In other words, we use these models much in the same way as we rely on the counting algorithms used to aggregate lexical frequency statistics: in both cases we are interested in the computed statistic (e.g., lexical surprisal or entropy, or lexical frequency) but we do not want to make any cognitive claim about the underlying algorithm that we happened to use to compute this statistic (e.g., GPT-2 for lexical surprisal, or a counting algorithm for lexical frequency). For more details on the exact choice, and relation to alternative metrics (e.g., GPT-3 or cloze probabilities) see Methods and Discussion.

### Prediction and Preview Increase Skipping Rates and Reduce Reading Times

We first asked whether our formalisations allowed us to observe the expected effects of prediction and preview, while statistically controlling for other explanatory variables. This was done by performing a multiple regression analysis, and statistically testing whether the coefficients were in the expected direction. Word skipping was modelled with a logistic regression, reading times (gaze durations) were predicted using ordinary least squares regression. Because the decisions of whether to skip and how long to fixate a word are made at different moments, when different types of information are available, we modeled each separately with a different set of explanatory variables. But in both cases, for inference on the coefficients, we considered the full model (variables motivated and detailed below; see Tables A.1 and A.2 for a tabular overview of all variables).

As expected, we found in all datasets that words were more likely to be skipped if there was more information available from the linguistic prediction (Bootstrap: Dundee, *p* = 0.023; GECO, *p* = 0.034; Provo, *p* < 10^−5^) and/or the parafoveal preview (Bootstrap: Dundee, *p* = 4 × 10^−5^; GECO, *p* < 10^−5^; Provo, *p* < 10^−5^). Similarly, reading times were reduced for words that were more predictable (all *p*’s < 3.2 × 10^−4^) or more identifiable from the parafovea (all *p*’s < 4 × 10^−5^). Together this confirms that our model-based approach can capture the expected effects of both prediction (Clifton et al., 2016) and preview (Schotter et al., 2012) in natural reading, while statistically controlling for other variables.

### Word Skipping is Largely Independent of Online Lexical Processing

After confirming that prediction and preview had a statistically significant influence on word skipping and reading times, we went on to assess their relative explanatory power. That is, we asked how important these factors were, by examining how much variance was explained by each. To this end, we grouped the variables from the full regression model into different types of explanations, and assessed how well each type accounted for the data, in terms of the unique and overlapping amount of variation explained by each explanation. This in turn was measured by the cross-validated *R*^2^ for reading times, and $R_{McF}^{2}$ for skipping, which both quantify the proportion of variation explained (see Methods).

For skipping, we considered three explanations. First, a word might be skipped *purely* because it could be predicted from context—that is, purely as a function of the amount of information about word identity conveyed by the prediction. Secondly, a word might be skipped because its identity could be gleaned from a parafoveal preview—that is, purely as a function of the amount of information about word identity conveyed by the preview. Finally, a word might be skipped simply because it is so short or so close to the prior fixation location that an autonomously generated saccade will likely overshoot it, irrespective of its linguistic properties—in other words, purely as a function of length and eccentricity. Note that we did not include often-used lexical attributes like frequency to predict skipping, because using attributes of word_*n*+1_ already pre-supposes parafoveal identification. Moreover, to the extent that a lexical attribute like frequency might influence a word’s parafoveal identifiability, this should already be captured by the parafoveal entropy (see Figure A.3 and Methods for more details).

**Figure 2.**
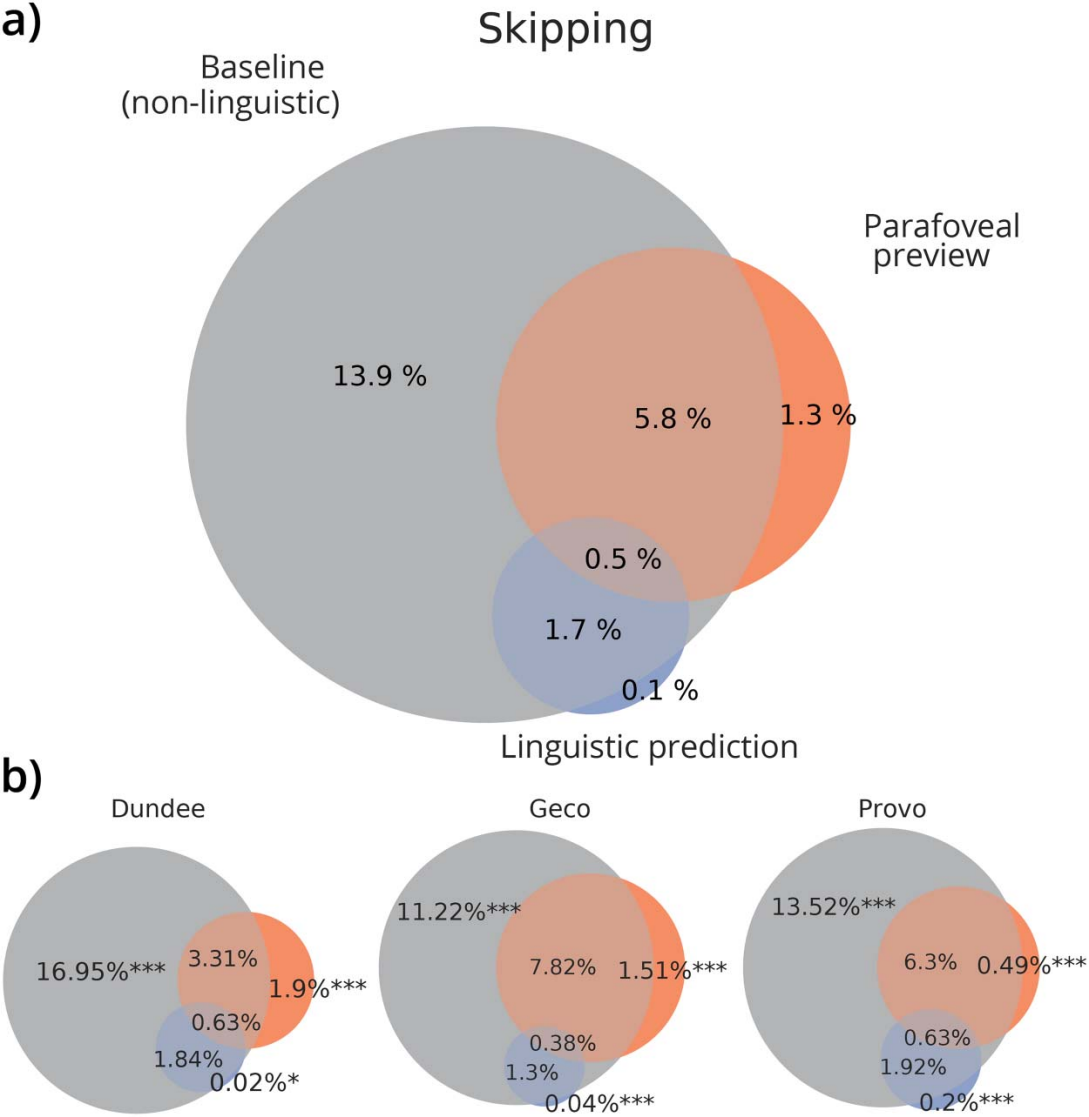
**Variation in skipping explained by predictive, parafoveal and autonomous oculomotor processing.** (A) Proportions of cross-validated variation explained by prediction (blue), preview (orange) oculomotor baseline (grey) and their overlap; averaged across datasets (each dataset weighted equally). (B) Variation partitions for each individual dataset, including statistical significance of variation uniquely explained by predictive, parafoveal or oculomotor processing. Stars indicate significance-levels of the cross-validated unique variation explained (bootstrap *t*-test against zero): *p* < 0.05 (*), *p* < 0.05 (**), *p* < 0.001 (***) For results of individual participants, and their consistency, see Figure A.9.

For each word, we thus modelled the probability of skipping either as a function of prediction, preview, or oculomotor information (i.e., eccentricity and length), or by any combination of the three. Then we partitioned the unique and shared cross-validated variation explained by each account. Strikingly, this revealed that the overwhelming majority of explained skipping variation (94%) could be accounted for by the oculomotor baseline that consisted just of eccentricity and length (Figure 2). Moreover, the majority of the variation was *only* explained by the baseline, which explained 10 times more unique variation than prediction and preview combined. There was a large degree of overlap between preview and the oculomotor baseline, which is unsurprising since a word’s identifiability decreases as a function of its eccentricity and length. Interestingly, there was even more overlap between the prediction and baseline model: almost all skipping variation that could be explained by contextual constraint could be equally well explained by the oculomotor baseline factors.

Importantly, while the contribution of prediction and preview was small, it was significant both for prediction (Dundee: 0.015% bootstrap 95CI: 0.003–0.029%; bootstrap *t*-test compared to zero, *p* = 0.014; Geco: 0.039%, 95CI: 0.018–0.065%; *p* = 0.0001; Provo: 0.20%; 95CI: 0.14–0.28%, *p* < 10^−5^) and preview (Dundee: 2.14%, 95CI: 1.66–2.60%; *p* < 10^−5^; Geco: 1.71%, 95CI: 1.20–2.29%, *p* < 10^−5^; Provo: 0.56%, 95CI: 0.36–0.79%, *p* < 10^−5^), confirming that both factors do affect skipping. Crucially however, the vast majority of skipping that could be explained by either prediction or preview was equally well explained by the more low-level and computationally frugal oculomotor model—which also explained much more of the skipping data overall. This challenges the idea that word identification is the main driver behind skipping, instead pointing to a more low-level, computationally simpler strategy.

What might this simpler strategy be? One possibility is a ‘blind’ random walk: generating saccades of some average length, plus oculomotor noise. However, we find that saccades are tailored to word length and exhibit a well-known preferred landing position, slightly left to a word’s centre (see Figure A.8; compare McConkie et al., 1988; Rayner, 1979). This suggests the decision of where to look next is not ‘blind’ but is based on a coarse low-level visual analysis of the parafovea, for instance conveying just the location of the next word ‘blob’ within a preferred range (i.e., skipping words too close or short; cf. Brysbaert et al., 2005; Deubel et al., 2000; Reilly & O’Regan, 1998). Presumably, such a simple strategy would on average sample visual input conveniently, yielding saccades large enough to read efficiently but small enough for comprehension to keep track. However, if such an ‘autopilot’ is indeed largely independent of online comprehension, one would expect it occasionally go out of step, such that a skipped word cannot be recognised or guessed, derailing comprehension. In line with suggestion, we find evidence for a compensation strategy. The probability that an initially skipped words is subsequently (regressively) fixated is significantly, inversely related to its parafoveal identifiability *before* skipping (see Figure A.10; logistic regression to prior parafoveal entropy: all *β*’s > 0.15; bootstrap test on coefficients: all *p*’s < 10^−5^). Together, this suggests that initial skipping decisions are primarily driven by a low-level oculomotor ‘autopilot’, which is kept in line with online comprehension by correcting saccades that outrun word recognition (much in line with the suggestions by Brysbaert et al., 2005).

### Reading Times are Strongly Modulated by Lexical Processing Difficulty

For reading times (defined as gaze durations, so considering foveal reading time only), we similarly considered three broad explanations. First, a word might be read faster because it was predictable from the preceding context, which we formalised via lexical surprisal. Second, a word might be read faster if it could already be partly identified from the parafoveal preview (before fixation). This informativeness of the preview was again formalised via the parafoveal preview entropy. Finally, a word might be read faster due to non-contextual attributes of the fixated word itself, such as frequency or word-class or the viewing position. This last explanatory factor functioned as a baseline that captured key non-contextual attributes, both linguistic and non-linguistic (see Methods).

In all datasets, we again found that all explanations accounted for some unique variation: prediction (Dundee: 0.80% bootstrap 95CI: 0.55–1.09%, bootstrap *t*-test compared to zero: *p* < 6^−5^; Geco: 0.68%, 95CI: 0.55–0.83%; *p* = 0.0001; Provo: 0.35%, 95CI: 0.20–0.43%, *p* < 10^−5^), preview (Dundee: 1.91%, 95CI: 1.00–3.14%, *p* = 0.00012; Geco: 1.59%, 95CI: 0.96–2.30%, *p* = 5 × 10^−5^; Provo: 0.93%, 95CI: 0.70–1.98%, *p* < 10^−5^) and the non-contextual word attributes (Dundee: 8.06%, 95CI: 5.84–10.32%, *p* = 5 × 10^−5^; Geco: 1.99%, 95CI: 1.32–2.81%, *p* < 10^−5^; Provo: 5.38%, 95CI: 4.48–6.83%, *p* < 10^−5^).

**Figure 3.**
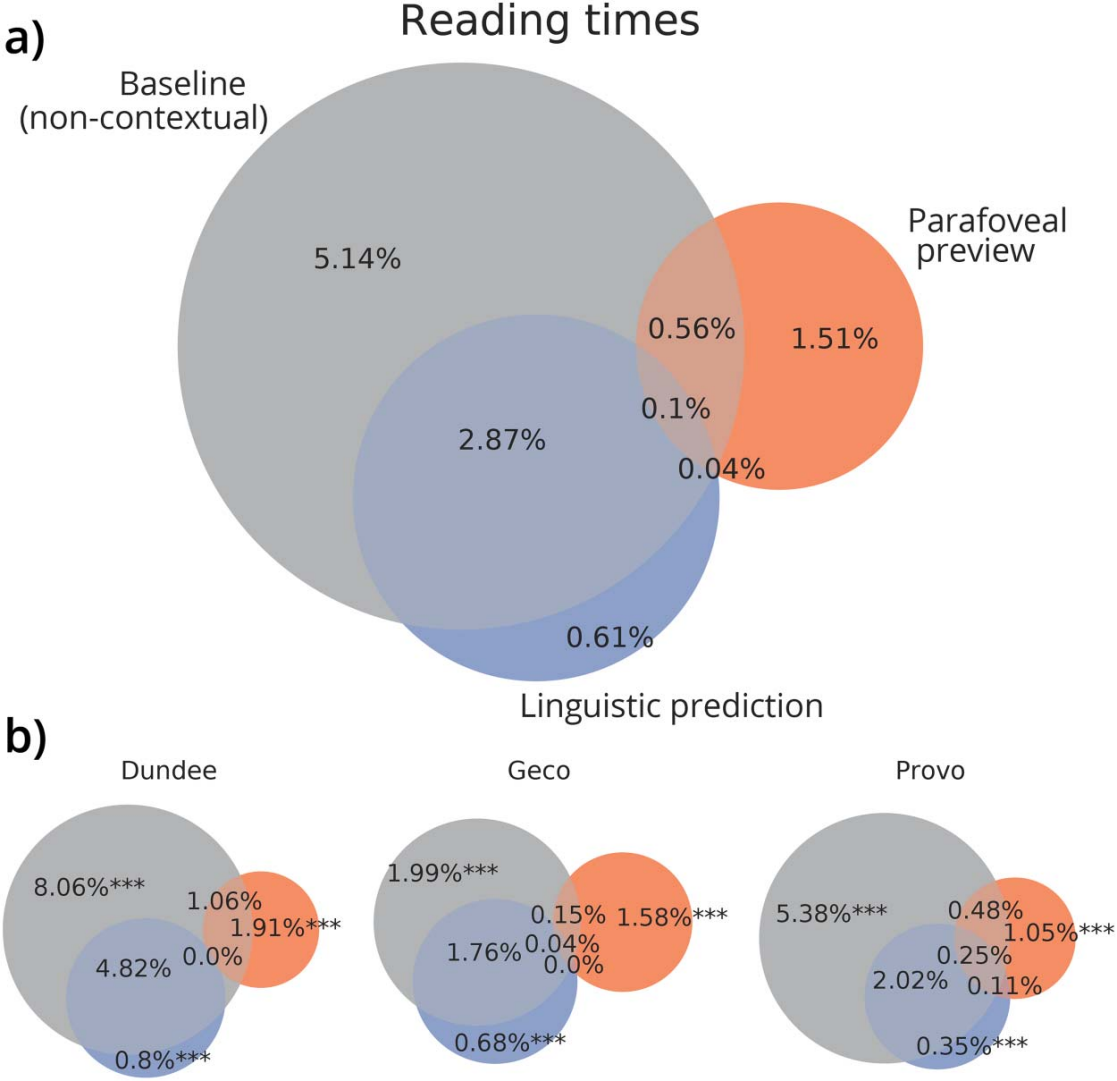
**Variation in reading times explained by predictive, parafoveal and non-contextual information.** (A) Grand average of partitions of cross-validated variance in reading times (indexed by gaze durations) across datasets (each dataset weighted equally) explained by non-contextual factors (grey), parafoveal preview (orange), and linguistic prediction (blue). (B) Variance partitions for each individual dataset, including statistical significance of the cross-validated variance explained uniquely by the predictive, parafoveal or non-contextual explanatory variables. Stars indicate significance levels of the cross-validated unique variance explained (bootstrap *t* test against zero): *p* < 0.05 (**), *p* < 0.001 (***). For results of individual participants, see Figure A.11. Note that the baseline model here both lexical attributes (e.g., frequency) and oculomotor factors (relative viewing/landing position). For a direct contrast between lexical processing-based explanations and purely oculomotor explanations, see Figure 4.

The non-contextual baseline explained the most variance, which shows—unsurprisingly—that properties of the fixated word itself are more important than contextual factors in determining how long a word is fixated. Critically however, compared to skipping the *unique* contribution of prediction and preview was more than three times higher (see Figure 3). Specifically, while prediction and preview could only uniquely account for 6% of explained word skipping variation, they uniquely accounted for more than 18% of explained variation in reading times. This suggests that while for skipping most explained variation can be accounted for by purely oculomotor variables, this is not the case for reading times.

**Figure 4.**
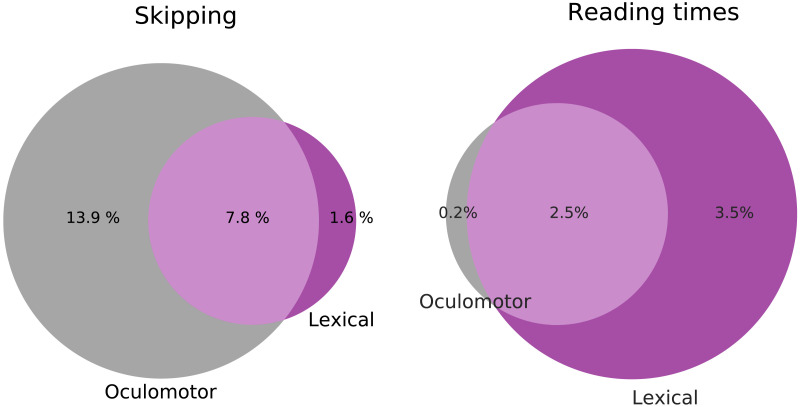
**Comparing oculomotor and lexical processing-based explanations for skipping and reading times.** Analysis with the same explanatory variables as Figures 2 and 3, grouped differently to directly contrast purely oculomotor explanatory variables and those that affect lexical processing ease (such as predictability, parafoveal identifiability, and lexical frequency; see Methods). Venn diagrams represent the proportions of unique and overlapping amount of explained variation (in *R*^2^ for reading times, and $R_{McF}^{2}$ for skipping) by each explanation (grand average across datasets). For partitions for individual datasets with statistics, see Figure A.12; for an alternative partitioning that includes word length in the oculomotor of reading times, see Figure A.13.

However, this comparison (between oculomotor and lexical processing based accounts) difficult to make based on the Figures 2 and 3 alone. This is because in the reading times analysis, the baseline model contained both oculomotor (i.e., viewing position) and lexical factors (notably lexical frequency). Therefore, we performed an additional analysis, grouping the explanatory variables differently to contrast purely oculomotor explanatory variables versus variables affecting lexical processing ease (such as predictability, parafoveal identifiability, and lexical frequency; see Tables A.3 and A.4). This shows that for skipping, purely oculomotor explanations can account for much more than a lexical processing-based explanation—but for reading times, it is exactly the other way around (Figure 4). Note that in Figure 4, the oculomotor model for reading times only contains variables quantifying viewing/landing position, because this is the primary oculomotor explanation for reading time differences (O’Regan, 1980, 1992; Vitu et al., 1990). If we also include word length in the oculomotor model for reading times, there is much more overlapping variance explained by the lexical and oculomotor model, presumably due to the correlation between word length and (log)frequency, which may inflate the importance of the oculomotor account (see Figure A.13). However, even with this potentially inflated estimate, the overall dissociation persists: if we compare the ratios unique variation explained by oculomotor vs. lexical processing-based models, there is still more than a 30-fold difference between the skipping and reading times analysis (in Figure A.13A). Together, this supports that for skipping, most explained variation is captured by purely oculomotor rather than lexical processing-based explanations, whereas for reading times, it is the other way around.

### Model-Based Estimates of Naturalistic Prediction and Preview Benefits Match Experimental Effect Sizes

The reading times results confirm that reading times are highly sensitive to factors influencing a word’s lexical processing ease, including contextual factors like linguistic and parafoveal context. This is in line with the scientific consensus and decades of experimental research on eye movements in reading (Rayner, 2009). But how well do our model-based, correlational results compare exactly to findings from the experimental literature?

To directly address this question, we quantitatively derived, for each participant, the effect size of two well-established effects that would be expected to be obtained if we would conduct a well-controlled factorial experiment. While we did not actually perform such a factorial experiment, we can derive this from the regression model, because we quantitatively estimated how much additional information from either prediction or preview (in bits) reduced reading times (in milliseconds). Therefore, the regression analyses allows us to estimate the expected difference in reading times for words that are expected vs. unexpected (predictability benefit; Rayner & Well, 1996; Staub, 2015) or have valid vs. invalid preview (i.e., preview benefit; Schotter et al., 2012).

**Figure 5.**
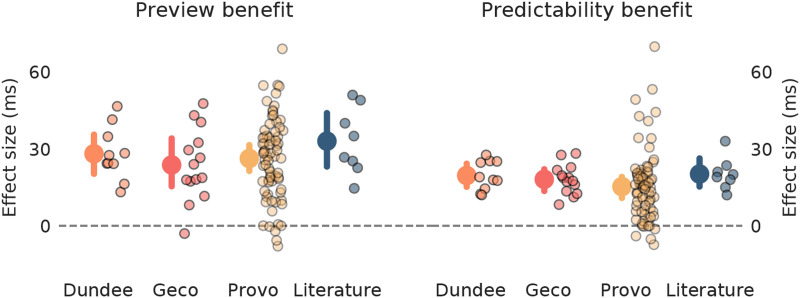
**Model-derived effect sizes match experimentally observed effect sizes.** Preview (left) and predictability benefits (right) inferred from our analysis of each dataset, and observed in a sample of studies (see Table A.5). In this analysis, preview benefit was derived from the regression model as the expected difference in gaze duration after a preview of average informativeness versus after no preview at all. Predictability benefit was defined as the difference in gaze duration for high versus low probability words; ‘high’ and ‘low’ were defined by subdividing the cloze probabilities from Provo into equal thirds of ‘low’, ‘medium’ and ‘high’ probability (see Methods). In each plot, small dots with dark edges represent either individual subjects within one dataset or individual studies in the sample of the literature; larger dots with error bars represent the mean effect across individuals or studies, plus the bootstrapped 99%CI.

Interestingly, the model-derived effect sizes are very well in line with those observed in experimental studies (see Figure 5). This suggests that our analysis does not strongly underfit or otherwise underestimate the effect of prediction or preview. Moreover, it shows that the effect sizes, which are well-established in controlled designs, generalise to natural reading. This last point is especially interesting for the preview benefit, because it implies that the effect can be largely explained in terms of parafoveal lexical identifiability (Pan et al., 2021; Rayner, 2009), and that other factors such as low-level visual ‘preprocessing’, or interference between the (invalid) parafoveal percept and foveal percept, may only play a minor role (cf. Reichle et al., 2003; Schotter et al., 2012).

### No Integration of Prediction and Preview

So far, we have treated prediction and preview as being independent. However, it might be that these processes, while using different information, are integrated—such that a word is parafoveally more identifiable when it is *also* more predictable in context. Bayesian probability theory proposes an elegant and mathematically optimal way to integrate these sources of information: the prediction of the next word could be incorporated as a prior in perceptual inference. Such a contextual prior fits into hierarchical Bayesian models of vision (Lee & Mumford, 2003), and has been observed in speech perception, where a contextual prior guides the recognition of words from a partial sequence of phonemes (Brodbeck et al., 2022; Heilbron et al., 2022). Does such a prior also guide word recognition in reading, based on a partial parafoveal percept?

**Figure 6.**
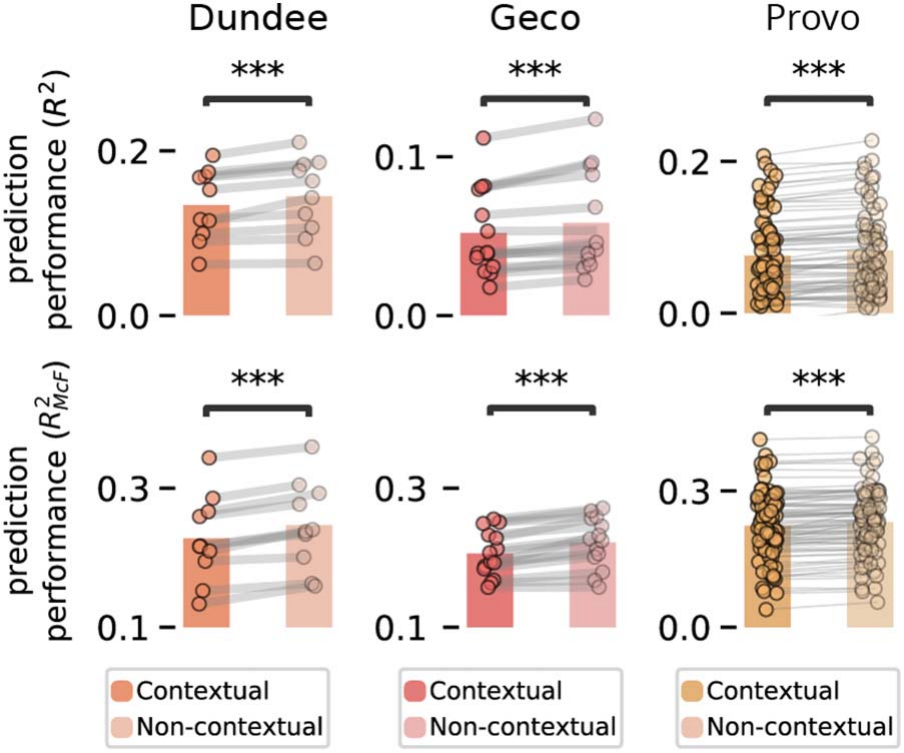
**Evidence against bayesian integration of linguistic prediction and parafoveal preview.** Cross-validated prediction performance of the full reading times (top) and skipping (bottom) model (including all variables), equipped with parafoveal preview information either from the contextual observer or from the non-contextual observer. Dots with connecting lines indicate participants; stars indicate significance: *p* < 0.001 (***).

To test this, we recomputed the parafoveal identifiability of each word for each participant, but now with an ideal observer using the prediction from GPT-2 as a prior. As expected, bayesian integration enhanced perceptual inference: on average, the observer using linguistic prediction as a prior extracted more information from the preview (± 6.25 bits) than the observer not taking the prediction into account (± 4.30 bits; *T*_1.39×10^6^_ = 1.35 × 10^11^, *p* ≈ 0). Interestingly however, it provided a worse fit to the human reading data. This was established by comparing two versions of the full regression model: one with parafoveal entropy from the (theoretically superior) contextual ideal observer and one from the non-contextual ideal observer. In all datasets both skipping and reading times were better explained by a model including parafoveal identifiability from the non-contextual observer (skipping: all *p*’s < 10^−5^; reading times: *p*’s < 10^−5^; see Figure 6). This replicates Duan and Bicknell (2020), who performed a similar analysis comparing a contextual (5-gram) and non-contextual prior in natural reading (Duan & Bicknell, 2020). Our findings replicate and significantly extend their findings, since Duan and Bicknell (2020) only analysed skipping in the Dundee corpus. Our analysis not only investigates additional datasets but it finds exactly the same result for reading times (for which the importance of both prediction and preview is decidedly larger).

Together, this suggests while both linguistic prediction and parafoveal preview influence online reading behaviour, the two sources of information are not integrated, but instead operate independently—highlighting a remarkable sub-optimality in reading.

## DISCUSSION

Eye movements during reading are highly variable. Across three large datasets, we assessed the relative importance of different explanations for this variability. In particular, we quantified the importance of two major contextual determinants of a word’s lexical processing difficulty—linguistic prediction and parafoveal preview—and compared such lexical processing-based explanations to alternative (non-linguistic) explanations. This revealed a stark dissociation: for word skipping, a simple low-level oculomotor model (using just word length and distance to prior fixation location) could account for much more (unique) variation than lexical processing-based explanations, whereas for reading times, it was exactly the other way around. Interestingly, preview effects were best captured by a non-contextual observer, suggesting that while readers use both linguistic prediction and preview, these do not appear to be integrated on-line. Together, the results underscore the dissociation between skipping and reading times, and show that for word skipping, the link between eye movements and cognition is less direct than commonly thought.

Our results on skipping strongly support the earlier findings and theoretical perspective by Brysbaert et al. (2005). They analysed effect sizes from studies on skipping and found a disproportionately large effect of length, compared to proxies of processing-difficulty like frequency and predictability. We significantly extend their findings by modelling skipping itself (rather than effect sizes from studies) and making a direct link to processing mechanisms. For instance, based on their analysis it was unclear how much of the length effect could be attributed to the lower visibility of longer words—that is, how much of the length effect may be an identifiability effect (Brysbaert et al., 2005, p. 19). We show that length and eccentricity alone explained three times as much variation as parafoveal identifiability—and that most of the variation explained by identifiability was equally well explained by length and eccentricity. This demonstrates that length and eccentricity themselves—not just to the extent they reduce identifiability—are key drivers of skipping.

This conclusion challenges dominant, cognitive models of eye movements, which describe lexical identification as the primary driver behind skipping (Engbert & Kliegl, 2003; Engbert et al., 2005; Reichle et al., 2003). Importantly, our results do not challenge predictive or parafoveal word identification itself. Rather, they challenge the notion that moment-to-moment decisions of whether to skip individual words are primarily driven by the recognition of those words. Instead, our results suggest a simpler strategy in which a coarse (e.g., dorsal stream) visual representation is used to reflexively select the next saccade target following the simple heuristic to move forward to the next word ‘blob’ within a certain range (see also Brysbaert et al., 2005; Deubel et al., 2000; Reilly & O’Regan, 1998).

Given that readers use both prediction and preview, why would they strongly affect reading times but hardly word skipping? We suggest this is because these different decisions—of *where* versus *how long* to fixate—are largely independent and are made at different moments (Findlay & Walker, 1999; Hanes & Schall, 1995; Schall & Cohen, 2011). Specifically, the decision of where to fixate—and hence whether to skip the next word—is made early in saccade programming, which can take 100–150 ms (Becker & Jürgens, 1979; Brysbaert et al., 2005; Hanes & Schall, 1995). Although the exact sequence of operations leading to a saccade remains debated, given that readers on average only look some 250 ms at a word, it is clear that skipping decisions are made under strong time constraints, especially given the lower processing rate of parafoveal information. We suggest that the brain meets this constraint by resorting to a computationally frugal ‘move forward’ policy. How long to fixate, by contrast, depends on saccade *initiation*. This process is separate from target selection, as indicated by physiological evidence that variation in target selection time only weakly explains variation in initiation times, which are affected by more factors and can be adjusted later (Findlay & Walker, 1999; Schall & Cohen, 2011). This can allow initiation to be informed by foveal information, which is processed more rapidly and may thus more directly influence the decision to either keep dwelling or execute the saccade.

One simplifying assumption we made is that during natural reading, the relative importance of identification-related processes (like prediction and preview) and oculomotor processsing are relatively stable within a single reader. However, it might be that underneath the average, aggregate relative importance we estimated, there is variability between specific moments in a text, or even within a sentence, during which the relative importance might be quite different. One such moment could be sentence transitions, where due to end-of-sentence ‘wrap up’ effects (Andrews & Veldre, 2021; Just & Carpenter, 1980), the relative importance of for instance preview may be reduced. Here we did not treat sentence transitions (or other such moments) as special, but looking into the possibility of moment-to-moment variability in relative importance is an interesting avenue for future research.

A distinctive feature of our analysis is that we focus on a few sets of computationally explicit variables, each forming a coherent explanation, and quantify the shared and unique variation accounted for by each explanation. The advantage of this approach is interpretability. However, a limitation of the partitioning analysis is that it is not always possible to add all potentially statistically significant variables (or interactions) to the regression, because partitioning requires that each variable can be assigned to a single explanation. When this is not possible (e.g., when a variable is (indirectly) associated with multiple explanations) this requires making a decision. Either the variable is omitted and the regression may not capture all explainable variance. Alternatively, the variable is assigned to just one explanation, which may distort the results by inflating the importance of that explanation.

A primary example of a variable requiring such a decision is (log)-frequency in the context of skipping. Frequency is sometimes used to predict skipping, as a proxy for a word’s parafoveal identifiability. However, this relationship is indirect, and (log)frequency is also—and much more strongly—correlated with length, and is hence also associated with the oculomotor explanation. Therefore, if one uses frequency as a proxy for parafoveal identifiability, one may find apparent preview effects which are in fact length effects, and strongly overestimate preview importance (Brysbaert & Drieghe, 2003). To avoid such overestimates, especially because the effect of frequency on identifiability should already be captured by the Ideal Observer (see Figure A.3 and Methods), we did not include frequency in our skipping analysis, nor did we include any other attribute that sometimes used a ‘proxy’ for either prediction/constraint or preview. A conceptually related problem is posed by interactions between variables from different explanations, such as between prediction/preview entropy and oculomotor predictors. These are impossible to assign to a single explanation, and were hence excluded from the regression.

As a result, the regression model did not include some variables or interactions that were used by prior regression analyses of skipping or reading times. This means that our regression may leave some explainable variation unexplained, and that our importance estimates are specific to the variables we consider, and our modelling thereof. However, this is a limitation that we believe trades-off favourably against the advantages afforded by the analysis. In particular, because for both skipping and reading times (1) we included all the factors deemed most important by prior regression-based studies (e.g., Duan & Bicknell, 2020; Hahn & Keller, 2023; Kliegl et al., 2006); (2) the amount of overall (cross-validated) explained variation is in line with prior regression-based analyses (e.g., Duan & Bicknell, 2020; Kliegl et al., 2006); and (3) our model-based effect sizes of prediction and preview effects are well in line with those from the experimental literature, suggesting our modelling of prediction or preview does not significantly fail to capture major aspects of either (Figure 5). In sum, we therefore do not believe that our selective and computationally explicit regression analysis significantly underestimates major factors of importance, and we are optimistic that our analysis yielded the comprehensive, interpretable picture that we aimed for.

To quantify predictability (surprisal) and constraint (lexical entropy) we used a neural language model (GPT-2), instead of the more standard cloze procedure. The reason for this is that we are interested in natural texts, where many words will have relatively low predictability values (e.g., below *p* = 0.01) which are inherently difficult to estimate in a cloze task. Since the effect of word predictability is logarithmic (Shain et al., 2022; Smith & Levy, 2013) the differences between small probabilities (e.g., between *p* = 0.001 and *p* = 0.0001) can have non-negligible effects, which is why for natural texts language models are superior to cloze metrics to capture predictability effects. Since the PROVO corpus includes cloze probability for every word, we could confirm this empirically, finding that model-derived surprisal indeed predicts reading times much better (Figure A.1).

We used this specific language model (GPT-2) simply because it was among the best publicly available ones, and prior work demonstrates that better language models (measured in perplexity) also predict human reading behaviour better (Goodkind & Bicknell, 2018; Wilcox et al., 2020). This raises the question whether an even better model (e.g., GPT-3, GPT-4, GPT-5, etc.) could predict human behaviour even better, and whether this might change the results. However, we do not believe this is likely. First, compared to the increases in quality (decreases in perplexity) from ngrams to GPT, further model improvements will be very subtle when quantified in the aggregate, and since reading behaviour is itself a noisy metric it is not obvious if such improvements will have a measurable impact. Second, one recent study even suggested that models larger than GPT-2 (GPT-J and GPT-3) predicted reading slightly *worse*, perhaps due to super-human memorisation capacities (Shain et al., 2022). In short, we used GPT-2 simply because it is a strong measure of lexical predictability (in English). Our analyses do not depend on GPT-2 specifically, in the same way we do not believe the results would change if we would have used different (but similar quality) lexical frequency statistics.

One apparent complication is that the skipping and reading times analyses use different metrics for explained variation (*R*^2^ and $R_{McF}^{2}$). This is due to the difference between continuous and discrete variables. As a result, directly numerically comparing the two (e.g., interpreting 4% *R*^2^ as ‘less’ than 5% $R_{McF}^{2}$) is difficult. However, our comparisons between skipping and reading times are not based on such absolute, numerical comparisons. Instead, the conclusions only rely on comparing the relative importance of different explanations. In other words, comparing the relative size and overlap of Venn diagrams in Figures 2, 3 and 4 (and hence only directly comparing quantities of the same metric).

If one does look at absolute numerical values across Figures 2, 3 and 6, the *R*^2^ values of the reading times regression may seem rather small. This could indicate a poor fit, which would potentially undermine our claim that reading times are to a large degree explained by cognitive factors. However, we do not believe this is the case, since our *R*^2^’s for gaze durations are not lower than *R*^2^’s reported by other regression analyses in natural reading (e.g., Kliegl et al., 2006); and because we find effect sizes in line with the experimental literature (Figure 5). Therefore, we do not believe we overfit or underfit gaze durations. Instead, what the relatively low *R*^2^ values indicate, we suggest, is that gaze durations are inherently noisy; that only a limited amount of the variation is systematic variation. While this noisiness might be interesting in itself (e.g., reflecting an autonomous timer; Engbert et al., 2005), it is not of interest in this study, which focusses on systematic variation, and hence only on relative importance of different explanations, not on absolute *R*^2^ values.

A final notable finding is that preview was best explained by a non-contextual observer. This replicates the only other study that compared contextual and non-contextual models of preview (Duan & Bicknell, 2020). That study focussed on skipping; the fact that we obtain the same result for reading times and in different datasets strengthens the conclusion that context does not inform preview. This is also in line with a number of studies on skipping, suggesting no or a weak effect of contextual fit (Angele et al., 2014; Angele & Rayner, 2013; Hahn & Keller, 2023). However, it contradicts a possibly larger range of experimental studies on preview more broadly, that do find interactions between contextual constraint/prediction and preview (e.g., Balota et al., 1985; McClelland & O’Regan, 1981; Schotter et al., 2015; Veldre & Andrews, 2018). One explanation for this discrepancy stems from how the effect is measured. Experimental studies looked at the effect of context on the difference in reading time after valid versus invalid preview (Schotter et al., 2015; Veldre & Andrews, 2018). This may reveal a context effect not on recognition, but at a later stage (e.g., priming between context, preview and foveal word). Arguably, these yield different predictions. If context affects recognition it may allow identification of otherwise unidentifiable words. But if the interaction occurs later it may only *amplify* processing of recognisable words. Constructing a model that formally reconciles this discrepancy is an interesting challenge for future work.

Given that readers use both prediction and preview, why doesn’t contextual prediction *inform* preview? One explanation stems from time constraints imposed by eye movements. Given that readers on average only look some 250 ms at a word in which they have to recognise the foveal word and process the parafoveal percept, this perhaps leaves too little time to fully let the foveal word and context inform parafoveal preview. On the other hand, word recognition based on partial input also occurs in speech perception under significant time-constraints. But despite those constraints, sentence context does influence auditory word recognition (McClelland & Elman, 1986; Zwitserlood, 1989), a process best modelled by a contextual prior (i.e., the opposite of what we find here; Brodbeck et al., 2022; Heilbron et al., 2022). Therefore, rather than being related to time-constraints *per se*, it might be also related to the underlying circuitry. More precisely, the fact that contrary to auditory word recognition, visual word recognition is a laboriously acquired skill that occurs throughout areas in the visual system that are repurposed (not evolved) for reading (Dehaene, 2009; Yeatman & White, 2021). Therefore, global sentence context might be able to dynamically influence the recognition of speech sounds in temporal cortex, but not that of words in visual cortex; there, context effects might be confined to simpler, more local context, like lexical context effects on letter perception (Heilbron et al., 2020; Reicher, 1969; Wheeler, 1970; Woolnough et al., 2021).

In conclusion, we have found that two important contextual sources of information about next-word identity in reading, linguistic prediction and parafoveal preview, strongly drive variation in reading times, but hardly affect word skipping, which is largely based on low-level factors. Our results show that as readers, we do not always use all information available to us; and that we are, in a sense, of two minds: consulting complex inferences to decide how long to look at a word, while employing semi-mindless scanning routines to decide where to look next. It is striking that these disparate strategies operate mostly in harmony. Only occasionally they go out of step—then we notice that our eyes have moved too far and we have to look back, back to where our eyes left cognition behind.

## METHODS

We analysed eye-tracking data from three, big, naturalistic reading corpora, in which native English speakers read texts while eye-movement data was recorded (Cop et al., 2017; Kennedy, 2003; Luke & Christianson, 2016).

### Stimulus Materials

We considered the English-native portions of the Dundee, Geco and Provo corpora. The Dundee corpus comprises eye-movements from 10 native speakers from the UK (Kennedy, 2003), who read a total of 56.212 words across 20 long articles from The Independent newspaper. Secondly, the English portion of the Ghent Eye-tracking Corpus (Geco) (Cop et al., 2017) is a collection of eye movement data from 14 UK English speakers who each read Agathe Cristie’s *The Mysterious Affair at Styles* in full (54.364 words per participant). Lastly, the Provo corpus (Luke & Christianson, 2018) is a collection of eye movement data from 84 US English speakers, who each read a total of 55 paragraphs (extracted from diverse sources) for a total of 2.689 words.

### Eye Tracking Apparatus and Procedure

In all datasets, eye movements were recorded monocularly, by recording the right eye. In Geco and Provo, recordings were made using an EyeLink 1000 (SR Research, Canada) with a spatial resolution of 0.01° and a temporal resolution of 1000 Hz. For Dundee, a Dr. Bouis oculometer (Dr. Bouis, Kalsruhe, Germany), with a spatial resolution of <0.1° and a temporal resolution of 1000 Hz was used. To minimize head movement, the participant’s heads were stabilised with a chinrest (Geco, Provo) or a bite bar (Dundee). In each experiment, texts were presented in ‘screens’ with either five lines (Dundee) or one paragraph per screen (Geco and Provo), presented using a font size of 0.33° per character. Each screen began with a fixation mark (gaze trigger) that was replaced by the initial word when stable fixation was achieved. In all datasets, a 9-point calibration was performed prior to the recording. In the longer experiments, a recalibration was performed every three screens (Dundee) or either every 10 minutes or whenever the drift correction exceeded 0.5° (Geco). For Dundee and Provo, the order of different texts were randomized across participants. In Geco, the entire novel was read start to finish with breaks between each chapter, during which participants answered comprehension questions.

For each corpus the *x*, *y*-values per fixation position were converted into a word-by-word format. In Dundee, raw *x*, *y*-values were smoothed by rounding to single-character precision. In Geco and Provo, raw *x*, *y*-values for each within-word- or within-letter fixation were preserved and available for each word. Across the three data sets we redefined the bounding boxes around each word, such that they subtended the area between the first to the last character of the word, with the boundary set halfway to the neighbouring character (e.g., halfway the before and after the word). Punctuation before or after the word were left out, and words for which the bounding box was inconsistently defined were ignored. For distributions of saccade and fixation data, see Figures A.5–A.7.

### Language Model

Contextual predictions were formalised using a language model—a model computing the probability of each word given the preceding words. Here, we used GPT-2 (XL)—currently among the best publicly released English language models. GPT-2 is a transformer-based model, that in a single pass turns a sequence of tokens *U* = (*u*_1_, …, *u*_*k*_) into a sequence of conditional probabilities, (*p*(*u*_1_), *p*(*u*_2_|*u*_1_), …, *p*(*u*_*k*_|*u*_1_, …, *u*_*k*−1_)).

Roughly, this happens in three steps: first, an embedding encodes the sequence of symbolic tokens as a sequence of vectors, which are the first hidden state *h*_0_. Then, a stack of *n* transformer blocks each applies a series of operations resulting in a new set of hidden states *h*_*l*_, for each block *l*. Finally, a (log-)softmax layer is applied to compute (log-)probabilities over target tokens. In other words, the model can be summarised as follows:

$$h_{0}=UW_{e}+W_{p}$$ (1)

$$h_{l}=\text{transformer}\_\text{block}\;\left(h_{l-1}\right)\forall i\in \left[1,n\right]$$ (2)

$$P\left(u\right)=\text{softmax}\;\left(h_{n}W_{e}^{T}\right),$$ (3)

where *W*_*e*_ is the token embedding and *W*_*p*_ is the position embedding.

The key component of the transformer-block is *masked multi-headed self-attention*. This transforms a sequence of input vectors (**x**_1_, **x**_2_, …, **x**_*k*_) into a sequence of output vectors (**y**_1_, **y**_2_, …, **y**_*k*_). Fundamentally, each output vector **y**_*i*_ is simply a weighted average of the input vectors: **y**_*i*_ = $\sum_{j=1}^{k}$ *w*_*ij*_**x**_*j*_. Critically, the weight *w*_*i*,*j*_ is not a parameter, but is *derived* from a dot product between the input vectors $x_{i}^{T}$**x**_*j*_, passed through a softmax and scaled by a constant determined by the dimensionality *d*_*k*_: *w*_*ij*_ = (exp $x_{i}^{T}$**x**_*j*_/$\sum_{j=1}^{k}$ exp $x_{i}^{T}$**x**_*j*_)$\frac{1}{{\sqrt{d}}_{k}}$. Because this is done for each position, each input vector **x**_*i*_ is used in three ways: first, to derive the weights for its own output, **y**_*i*_ (as the *query*); second, to derive the weight for any other output **y**_*j*_ (as the *key*); finally, it is used in the weighted sum (as the *value*). Different linear transformations are applied to the vectors in each cases, resulting in Query, Key and Value matrices (*Q*, *K*, *V*). Putting this all together, we obtain:

$$\text{self}\_\text{attention}\;\left(Q,K,V\right)=\text{softmax}\;\left(\frac{QK^{T}}{\sqrt{d_{k}}}\right)V.$$ (4)

To be used as a language model, two elements are added. First, to make the operation position-sensitive, a position embedding *W*_*p*_ is added in the embedding step (Equation 1). Second, to enforce that the model only uses information from the past, attention from future vectors is masked out. To give the model more flexibility, each transformer block contains multiple instances (‘heads’) of the self-attention mechanisms from Equation 4. In total, GPT-2 (XL) contains *n* = 48 blocks, with 12 heads each; a dimensionality of *d* = 1600 and a context window of *k* = 1024, yielding a total of 1.5 × 10^9^ parameters. We used the PyTorch implementation of GPT-2 from the *Transformers* package (Wolf et al., 2020).

One complication of deriving word-probabilities from GPT-2 is that it doesn’t operate on words but on tokens. Tokens can be whole words (as with most common words) or sub-words. To derive word probabilities, we take the token probability for a single-token word, and the joint probability for words spanning multiple tokens, as is standard practice in psycholinguistics (Pimentel et al., 2022; Shain et al., 2022; Wilcox et al., 2020). However, because GPT marks word boundaries (i.e., spaces), at the *beginning* of a token, technically, the ‘end of word‘ decision is made at the next token. Defining word probabilities via (joint) constituent token probabilities doesn’t take this into account. Therefore, it will on average slightly over-estimate word probabilities (underestimate surprisal). However, this slight underestimation is not likely to affect any of our conclusions, since our regression analyses primarily depend on differences between *relative* predictabilities of different words, and since GPT-2, when used in this way, has been shown to result in probabilities that predict reading behaviour very well compared to other language models that do use whole-word tokenisation (Wilcox et al., 2020), and even compared to larger models like GPT-3 (Shain et al., 2022).

We chose GPT-2 because it is a high-quality language model (measured in perplexity on English texts), and better language models generally predict reading behaviour better (Goodkind & Bicknell, 2018; Wilcox et al., 2020). Our analysis does not depend on GPT-2 specifically, it could be switched with any similarly high-quality language model, much in the same way as we do believe our results to be specific to the exact lexical frequency statistics estimates we used (see Discussion).

### Ideal Observer

To compute parafoveal identifiability, we implemented an ideal observer based on the formalism by Duan and Bicknell (2020). This model formalises parafoveal word identification using Bayesian inference and builds on previous well-established ‘Bayesian Reader’ models (Bicknell & Levy, 2010; Norris, 2006, 2009). It computes the probability of the next word given a noisy percept by combining a prior over possible words with a likelihood of the noisy percept, given a word identity:

$$p\left(w|𝓘\right)\propto p\left(w\right)p\left(𝓘|w\right),$$ (5)

where 𝓘 represents the noisy visual input, and *w* represents a word identity. We considered two priors (see Figure 6): a non-contextual prior (the overall probability of words in English based on their frequency in Subtlex (Brysbaert & New, 2009), and a contextual prior based on GPT2 (see below). Below we describe how visual information is represented and perceptual inference is performed. For a graphical schematic of the model, see Figure A.2; for some distinctive simulations showing how the model captures key effects of linguistic and visual characteristics on word recognition, see Figure A.3.

#### Sampling Visual Information.

Like in other Bayesian Readers (Bicknell & Levy, 2010; Norris, 2006, 2009), noisy visual input is accumulated by sampling from a multivariate Gaussian which is centred on a one-hot ‘true’ letter vector—here represented in an uncased 26-dimensional encoding—with a diagonal covariance matrix Σ(*ε*) = *λ*(*ϵ*)^−1/2^*I*. The shape of Σ is thus scaled by the sensory quality *λ*(*ε*) for a letter at eccentricity *ε*. Sensory quality is computed as a function of the perceptual span: this uses a Gaussian integral based follows the perceptual span or processing rate function from the SWIFT model (Engbert et al., 2005). Specifically, for a letter at eccentricity *ε*, *λ* is given by the integral within the bounding box of the letter:

$$\lambda \left(\varepsilon \right)=\int_{\varepsilon -.5}^{\varepsilon +.5}\frac{1}{\sqrt{2\pi \sigma^{2}}}\exp\;\left(-\frac{x^{2}}{2\sigma^{2}}\right)dx,$$ (6)

which, following Bicknell and Levy (2010) and Duan and Bicknell (2020), is scaled by a scaling factor Λ. Unlike SWIFT, the Gaussian in Equation 6 is symmetric, since we only perform inference on information about the next word. By using one-hot encoding and a diagonal covariance matrix, the ideal observer ignores similarity structure between letters. This is clearly a simplification, but one with significant computational benefits; moreover, it is a simplification shared by all Bayesian Reader-like models (Bicknell & Levy, 2010; Duan & Bicknell, 2020; Norris, 2006), which can nonetheless capture many important aspects of visual word recognition and reading. To determine parameters Λ and *σ*, we performed a grid search on a subset of Dundee and Geco (see Figure A.4), resulting in Λ = 1 and *σ* = 3. Note that this *σ* value is close to the average *σ* value of SWIFT and (3.075) and corresponds well to prior literature on the size of the perceptual span (±15 characters; Bicknell & Levy, 2010; Engbert et al., 2005; Schotter et al., 2012).

#### Perceptual Inference.

Inference is performed over the full vocabulary. This is represented as a matrix which can be seen as a stack of word vectors, **y**_**1**_, **y**_**2**_, …, **y**_**v**_, obtained by concatenating the letter vectors. The vocabulary is thus a *V* × *d* matrix, with *V* the number of words in the vocabulary and *d* the dimensionality of the word vectors (determined by the length of the longest word: *d* = 26 × *l*_*max*_).

To perform inference, we use the belief-updating scheme from Duan and Bicknell (2020), in which the posterior at sample *t* is expressed as a (*V* − 1) dimensional log-odds vector **x**^(**t**)^, in which each entry $x_{i}^{\left(t\right)}$ represents the log-odds of **y**_**i**_ relative to the final word **y**_**v**_. In this formulation, the initial value of **x** is thus simply the prior log odds, $x_{i}^{\left(0\right)}$ = log *p*(*w*_*i*_) − log *p*(*w*_*v*_), and updating is done by summing prior log-odds and the log-odds likelihood. This procedure is repeated for *T* samples, each time taking the posterior of the previous timestep as the prior in the current timestep. Note that using log odds in this way avoids renormalization:

$$\begin{array}{l} x_{i}^{\left(t\right)}=\log\;\frac{p\left(w_{i}|{𝓘}^{\left(0,\ldots ,t\right)}\right)}{p\left(w_{v}|{𝓘}^{\left(0,\ldots ,t\right)}\right)}\\ \;=\log\;\frac{p\left(w_{i}|{𝓘}^{\left(0,\ldots ,t-1\right)}\right)p\left({𝓘}^{\left(t\right)}|w_{i}\right)}{p\left(w_{v}|{𝓘}^{\left(0,\ldots ,t-1\right)}\right)p\left({𝓘}^{\left(t\right)}|w_{v}\right)}\\ \;=\log\;\frac{p\left(w_{i}|{𝓘}^{\left(0,\ldots ,t-1\right)}\right)}{p\left(w_{v}|{𝓘}^{\left(0,\ldots ,t-1\right)}\right)}+\log\;\frac{p\left({𝓘}^{\left(t\right)}|w_{i}\right)}{p\left({𝓘}^{\left(t\right)}|w_{v}\right)}\\ \;=x_{i}^{\left(t-1\right)}+\Delta x_{i}^{\left(t\right)}. \end{array}$$ (7)

In other words, as visual sample 𝓘^(*t*)^ comes in, beliefs are updated by summing the prior log odds **x**^(*t*−1)^ and the log-odds likelihood of the new information **x**^(*t*)^.

For a given word *w*_*i*_, the log-odds likelihood of each new sample is the difference of two multivariate Gaussian log-likelihoods, one centred on **y**_*i*_ and one on the last vector **y***_v_*. This can be formulated as a linear transformation of 𝓘:

$$\begin{array}{l} \Delta x_{i}=\log\;p\left(𝓘|w_{i}\right)-\log\;p\left(𝓘|w_{v}\right)\\ \;=\log\;p\left(𝓘|𝒩\left(y_{i},\Sigma \right)\right)-\log\;p\left(𝓘|𝒩\left(y_{v},\Sigma \right)\right)\\ \;=\left[-\frac{1}{2}{\left(𝓘-y_{i}\right)}^{T}\Sigma^{-1}\left(𝓘-y_{i}\right)\right]-\\ \;\left[-\frac{1}{2}{\left(𝓘-y_{v}\right)}^{T}\Sigma^{-1}\left(𝓘-y_{v}\right)\right]\\ \;=\frac{y_{v}^{T}\Sigma^{-1}y_{v}-y_{i}^{T}\Sigma^{-1}y_{i}}{2}+{\left(y_{i}-y_{v}\right)}^{T}\Sigma^{-1}𝓘, \end{array}$$ (8)

which implies that updating can be implemented by sampling from a multivariate normal. To perform inference on a given word, we performed this sampling scheme until convergence (using *T* = 50), and then transformed the posterior log-odds into the log posterior, from which we computed the Shannon entropy as a metric of parafoveal identifiability.

To compute the parafoveal entropy for each word in the corpus, we make the simplifying assumption that parafoveal preview only occurs during the last fixation prior to a saccade, thus computing the entropy as a function of the word itself and its distance to the last fixation location within the previously fixated word (which is not always the previous word). Because this distance is different for each participant, it was computed separately for each word, for each participant. Moreover, because the inference scheme is based on sampling, we repeated it 3 times, and averaged these to compute the posterior entropy of the word. The amount of information obtained from the preview is then simply the difference between prior and posterior entropy.

The ideal observer was implemented in custom Python code, and can be found in the data sharing collection (see below).

### Contextual vs. Non-Contextual Prior

We considered two observers: one with a non-contextual prior capturing the overall probability of a word in a language, and with a contextual prior, capturing the contextual probability of a word in a specific context. For the non-contextual prior, we simply used lexical frequencies from which we computed the (log)-odds prior used in Equation 7. For the contextual prior, we derived the contextual prior from log-probabilities from GPT-2. This effectively involves constructing a new Bayesian model for each word, for each participant, in each dataset. To simplify this process, we did not take the full predicted distribution of GPT-2, but only the ‘nucleus’ of the top *k* predicted words with a cumulative probability of 0.95, and truncated the (less reliable) tail of the distribution. Further, we simply assumed that the rest of the tail was ‘flat’ and had a uniform probability. Since the prior odds can be derived from relative frequencies, we can think of the probabilities in the flat tail as having a ‘pseudocount’ of 1. If we similarly express the prior probabilities in the nucleus as implied ‘pseudofrequencies’, the cumulative implied nucleus frequency is then complementary to the length of the tail, which is simply the difference between the vocabulary size and nucleus size (*V* − *k*). As such, for word *i* in the text, we can express the nucleus as implied frequencies as follows:

$$\text{freq}s_{\psi }=P_{tr}\left(w^{\left(i\right)}|\text{context}\right)\frac{V-k}{1-\sum_{j=1}^{k}P\left(w_{j}^{\left(i\right)}|\text{context}\right)}$$ (9)

where *P*_*tr*_(*w*^(*i*)^ ∣ context) is the truncated lexical prediction, and *P*($w_{j}^{\left(i\right)}$ | context) is predicted probability that word *i* in the text is word *j* in the sorted vocabulary. Note that using this flat tail not only simplifies the computation, but also deals with the fact that the vocabulary of GPT-2 is smaller than that of the ideal observer—using this tail we can still use the full vocabulary (e.g., to capture orthographic uniqueness effects), while using 95% of the density from GPT-2.

### Data Selection

In our analyses, we focus on first-pass reading (i.e., progressive eye movements), analysing only those fixations or skips when none of the subsequent words have been fixated before. Moreover, we exclude return sweeps (i.e., line transitions), which are very different from within-line saccades, and hence excluded. We extensively preprocessed the corpora so that we could include as many words as possible. However, we had to impose some additional restrictions. Specifically we did not include words if they a) contained non-alphabetic characters; b) if they were adjacent to blinks; c) if the distance to the prior fixation location was more than 24 characters (±8); moreover, for the gaze duration we excluded d) words with implausibly short (< 70 ms) or long (> 900 ms) gaze durations. Criterion c) was chosen because some participants occasionally skipped long sequences of words, up to entire lines or more. Such ‘skipping’—indicated by saccades much larger than the perceptual span—is clearly different from the skipping of words during normal reading, and was therefore excluded. Note that these criteria are comparatively mild (cf. Duan & Bicknell, 2020; Smith & Levy, 2013), and leave approximately 1.1 million observations for the skipping analysis, and 593.000 reading times observations.

### Regression Models: Skipping

Skipping was modelled via logistic regression in scikit-learn (Pedregosa et al., 2011), with three sets of explanatory variables (or ’models’) each formalising a different explanation for why a word might be skipped.

First, a word might be skipped because it could be confidently predicted from context. We formalise this via *linguistic entropy*, quantifying the information conveyed by the prediction from GPT-2. We used entropy, not (log) probability, because using the next word’s probability directly would presuppose that the word is identified, undermining the dissociation of prediction and preview. By contrast, prior entropy specifically probes the information available from prediction only.

Secondly, a word might be skipped because it could be identified from a parafoveal preview. This was formalised via parafoveal entropy, which quantifies the parafoveal preview uncertainty (or, inversely, the amount of information conveyed by the preview). This is a complex function integrating low-level visual (e.g., decreasing visibility as a function of eccentricity) and higher-level information (e.g., frequency or orthographic effects) and their interaction (see Figure A.3). Here, too we did not use lexical features (e.g., frequency) of the next word to model skipping directly, as this presupposes that the word is identified; and to the extent that these factors are expected to influence identifiability, this is already captured by the parafoveal entropy (Figure A.3).

Finally, a word might be skipped simply because it is too short and/or too close to the prior fixation location, such that a fixation of average length would overshoot the word. This autonomous oculomotor account was formalised by modelling skipping probability purely as a function of a word’s length and its distance to the previous fixation location.

Note that these explanations are not mutually exclusive, so we also evaluated their combinations (see below).

### Regression Models: Reading Time

As an index of reading time, we analysed first-pass *gaze duration*, the sum of a word’s first-pass fixation durations. We analyse gaze durations as they arguably most comprehensively reflect how long a word is looked at, and are the focus of similar model-based analyses of contextual effects in reading (Goodkind & Bicknell, 2018; Smith & Levy, 2013). For reading times, we used linear regression, and again considered three sets of explanatory variables, each formalising a different kind of explanation.

First, a word may be read more slowly because it is unexpected in context. We formalised this using surprisal −log(*p*), a metric of a word’s unexpectedness—or how much information is conveyed by a word’s identity in light of a prior expectation about the identity. To capture spillover (Rayner et al., 2006; Smith & Levy, 2013) we included not just the surprisal of the current word, but also that of the previous two words.

Secondly, a word might be read more slowly because it was difficult to discern from the parafoveal preview. This was formalised using the parafoveal entropy (see above).

Finally, a word might be read more slowly because of non-contextual factors of the word itself. This is an aggregate baseline explanation, aimed to capture all relevant non-contextual word attributes, which we contrast to the two major contextual sources of information about a word identity that might affect reading times (prediction and preview). We included word class, length, log-frequency, and the relative landing position (quantified as the distance to word centre, both in fraction and in characters). For log-frequency we used the UK or US version of SUBTLEX depending on the corpus and included the log-frequency of the past two words to capture spillover effects.

The full model was defined as the joint of all models. For a tabular overview of all explanatory variables, see Tables A.1–A.3.

### Model Evaluation

We compared the ability of each model to account for the variation in the data by probing prediction performance in a 10-fold cross-validation scheme, in which we quantified how much of the observed variation in skipping rates and gaze durations could be explained.

For reading times, we did this using the coefficient of determination, defined via the ratio of residual and total sum of squares: *R*^2^ = 1 − $\frac{SS_{res}}{SS_{tot}}$. The ratio $\frac{SS_{res}}{SS_{tot}}$ relates the error of the model (*SS*_*res*_) to the error of a ‘null’ model predicting just the mean (*SS*_*tot*_), and gives the variance explained. For skipping, we use a tightly related metric, the McFadden *R*^2^. Like the *R*^2^ it is computed by comparing the error of the model to the error of a null model with only an intercept: $R_{McF}^{2}$ = 1 − $\frac{L_{M}}{L_{\text{null}}}$, where *L* indicates the loss.

While *R*^2^ and $R_{McF}^{2}$ are not identical, they are formally tightly related—critically, both are zero when the prediction is constant (no variation explained) and go towards one proportionally as the error decreases to zero (i.e., towards all variation explained). Note that in a cross-validated setting, both metrics can become negative when prediction of the model is worse than the prediction of a constant null-model.

### Variation Partitioning

To assess relative importance, we used variation partitioning to estimate how much explained variation could be attributed to each set of explanatory variables. This is also known as *variance* partitioning, as it is originally based on partitioning sums of squares; here we use the more general term ‘variation’ following Legendre (2008).

Variation partitioning builds on the insight that when two (groups of) explanatory variables (*A* and *B*) both explain some variation in the data *y*, and *A* and *B* are independent, then variation explained by combining *A* and *B* will be approximately additive. By contrast, when *A* and *B* are fully redundant (e.g., when *B* only has an *apparent* effect on *y* through its correlation with *A*), then a model combining *A* and *B* will not explain more than the two alone. Following de Heer et al. (2017), we generalise this logic to up to three (sets of) explanatory variables, by testing each individually and all combinations, and using set theory notation and graphical representation for its simplicity and clarity.

A two-way partition of two sets of explanatory variables (Figures 4, A.12, A.13) involves (*A* and *B*) fitting three models: two partial models with their features alone (*A* and *B*), and a joint model with both (*A* ∪ *B*). The unique variation explained by either A (A*) is derived via the difference between the partial models and the joint model:

$$\begin{array}{l} A^{*}=A\setminus B=A\cup B-B\\ B^{*}=B\setminus A=A\cup B-A \end{array}$$ (10)

And the intersection is derived from the joint model and sum of the partial models:

A∩B=A+B−A∪B (11)

For three groups of explanatory variables (*A*, *B*, and *C*), the situation is a bit more complex. We first evaluate each separately and all combinations, resulting in 7 models:

A,B,C,A∪B,A∪C,B∪C,A∪B∪C.

From these 7 models we obtain 7 ‘empirical’ scores (of variation explained), from which we derive the 7 ‘theoretical‘ partitions: 4 overlap partitions and 3 unique partitions. The first overlap partition is the variation explained by all models, which we can derive as:

A∩B∩C=A∪B∪C+A+B+C−A∪B−A∪C−B∪C. (12)

The next three overlap partitions contain all pairwise intersections of models that did not include the other model:

$$\begin{array}{l} \left(A\cap B\right)\setminus C=A+B-A\cup B-A\cap B\cap C\\ \left(A\cap C\right)\setminus B=A+C-A\cup C-A\cap B\cap C\\ \left(B\cap C\right)\setminus A=B+C-B\cup C-A\cap B\cap C. \end{array}$$ (13)

The last three partitions are those explained exclusively by each model. This is the relative complement: the partition unique to *A* is the relative complement of BC: *BC*^*RC*^. For simplicity we also use a star notation, indicating the unique partition of *A* as A*. These are derived as follows:

$$\begin{array}{l} A^{*}=BC^{RC}=A\cup B\cup C-B\cup C\\ B^{*}=AC^{RC}=A\cup B\cup C-A\cup C\\ C^{*}=AB^{RC}=A\cup B\cup C-A\cup B. \end{array}$$ (14)

Note that, in the cross-validated setting, the results can become paradoxical and depart from what is possible in classical statistical theory, such as partitioning sums of squares. For instance, due to over-fitting, a model that combines multiple EVs could explain *less* variance than all of the EVs alone, in which case some partitions would become negative. However, following de Heer et al. (2017), we believe that the advantages of using cross-validation outweigh the risk of potentially paradoxical results in some subjects. Partitioning was carried out for each subject, allowing to statistically assess whether the additional variation explained by a given model was significant. On average, none of the partitions were paradoxical.

### Simulating Effect Sizes

Regression-based preview benefits were defined as the expected difference in gaze duration after a preview of average informativeness versus after no preview at all. This best corresponds to an experiment in which the preceding preview was masked (e.g., XXXX) rather than invalid (see Discussion). To compute this we compared the took the difference in parafoveal entropy between an average preview and the prior entropy. Because we standardised our explanatory variables, this was transformed to subject-specific z-scores and then multiplied by the regression weights to obtain an expected effect size.

For the predictability benefit, we computed the expected difference in gaze duration between ‘high’ and ‘low’ probability words. ‘High’ and ‘low’ was empirically defined based on the human-normed cloze probabilities in Provo (using the OrthoMatchModel definition for additional granularity; Luke & Christianson, 2018), which we divided into thirds using percentiles. The resulting cutoff points (low < 0.02; high > 0.25) were log-transformed, applied to the surprisal values from GPT-2, and multiplied by the weights to predict effect sizes. Note that these definitions of ‘low’ and ‘high’ may appear low compared to those in the literature—however, most studies collect cloze only for specific ‘target’ words in relatively predictable contexts, which biases the definition of ‘low’ vs. ‘high’ probability. By contrast, we analysed cloze probabilities for *all* words, yielding these values.

### Statistical Testing

Statistical testing was performed across participants within each dataset. Because two of the three corpora had a low number of participants (10 and 14 respectively) we used data-driven, non-analytical bootstrap *t*-tests, that involve resampling a null-distribution with zero mean (by removing the mean), counting across bootstraps how likely a *t*-value at least as extreme as the true *t*-value was to occur. Each test used at least 10^4^ bootstraps; *p* values were computed without assuming symmetry (equal-tail bootstrap; Rousselet et al., 2019). Confidence intervals (in the figures and text) also based on bootstrapping.

## ACKNOWLEDGMENTS

We thank Maria Barrett, Yunyan Duan, and Benedikt Ehinger for useful input and inspiring discussions during various stages of this project.

## FUNDING INFORMATION

This work was supported by The Netherlands Organisation for Scientific Research (NWO Research Talent grant to M.H.; NWO Vidi 452-13-016 to F.P.d.L.; Gravitation Program Grant Language in Interaction no. 024.001.006 to P.H.) and the European Union Horizon 2020 Program (ERC Starting Grant 678286, “Contextvision” to F.P.d.L.).

## AUTHOR CONTRIBUTIONS

Conceptualisation: MH. Data wrangling and preprocessing: JvH. Formal analysis: MH, JvH. Statistical analysis and visualisations: JvH, MH. Supervision: FPdL, PH. Initial draft: MH. Final draft: MH, JvH, PH, FPdL.

## DATA AND CODE AVAILABILITY STATEMENT

The Provo and Geco corpora are freely available (Cop et al. 2017; Luke & Christianson, 2018). All additional data and code needed to reproduce the results will be made public on the Donders Repository at https://doi.org/10.34973/kgm8-6z09.
