# Supplementary material for: Lexical Processing Strongly Affects Reading Times But Not Skipping During Natural Reading
Source: Open Mind (Camb). 2023 Oct 1;7:757–83. doi: 10.1162/opmi_a_00099 (PMC10575561; doi:10.1162/opmi_a_00099)
Supplement: Supplementary file 1 [file opmi-07-757-s001.pdf]

Supplemental Material: Heilbron, M., van Haren, J., Hagoort, P., & de Lange, F. P. (2023). Lexical Processing Strongly Affects Reading Times But Not Skipping During Natural Reading. *Open Mind: Discoveries in Cognitive Science.*
https://doi.org/10 .1162/opmi_a_00099

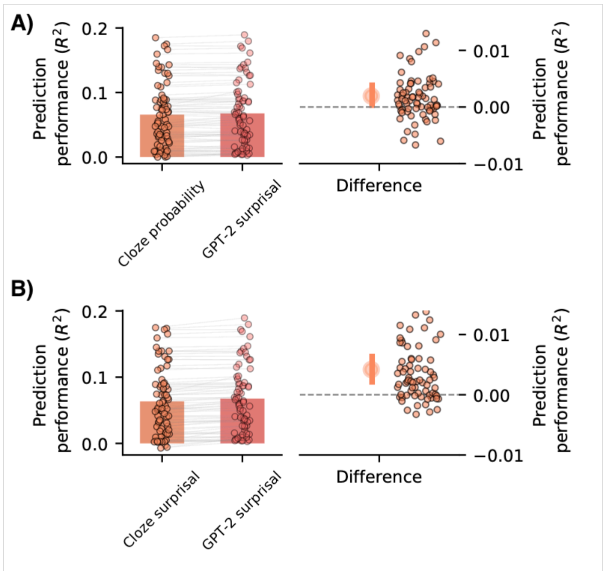

Figure A.1. GPT-2 predictabilities outperform cloze predictabilities. Result of a cross-validated model comparsion in the full reading times model, using either GPT-2-derived surprisal as the lexical predictability metric, or a cloze task derived probability (**A**) or log-probability (**B**) value, evaluated on provo, since this includes cloze-norm-derived probability values for each word. In both cases we used the full reading times model, similar to the model comparison in Figure 6. The regression model with GPT-2 predictability values performs much better (bootstrap: $P < 0.00001$), this is not surprising because the cloze probabilities are not sensitive to small probability values, and hence unable to distinguish between subtle differences in predictability (e.g. between 0.01 and 0.001 or 0.0001) which are known to be important for modelling predictability effects in human langauge processing (Shain et al., 2022; Smith & Levy, 2013). Hence, this analysis confirms that for word-by-word predictability estiamtes in natural texts, where consraint is generally low (Luke & Christianson, 2016), language model derived predictabilities are superior to cloze-task-derived probability estimates.

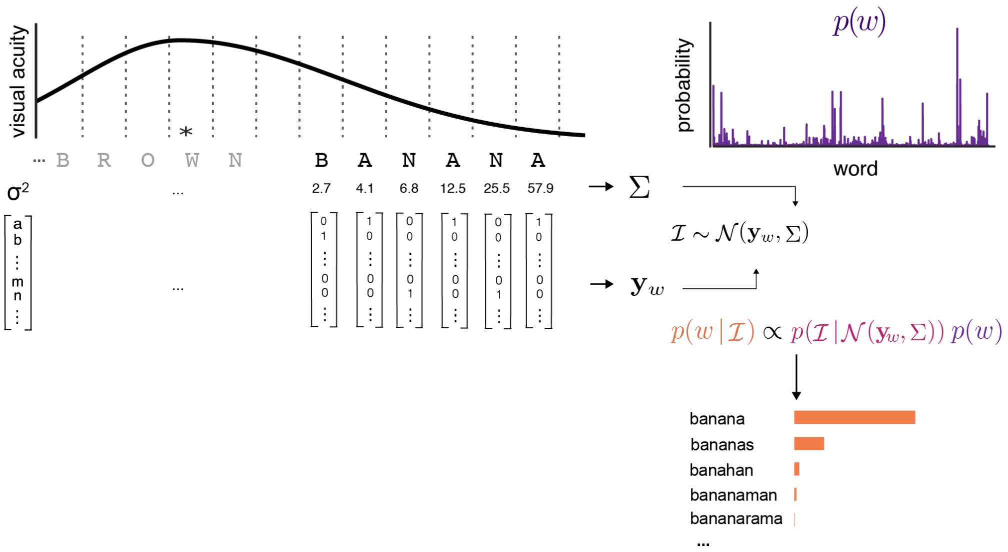

Figure A.2. Encoding and inference scheme of the ideal observer analysis. Visualisation of the Ideal Observer, following formulation in Duan and Bicknell (2020). A word at a given eccentricity is converted into a noisy visual percept, after which a posterior probability of the identity of the word given the noisy percept was computed using Bayesian inference. The uncertainty of this posterior (expressed in terms of Shannon entropy) was then used to quantify the expected uncertainty in the parafoveal percept – or, inversely, a word's *parafoveal identifiability*. In this scheme, words are represented as a concatenation of one-hot encoded letter vectors. Visual information ($\mathcal{I}$) is sampled from a multivariate Gaussian centred on the word vector $\mathbf{y}_w$ with a diagonal covariance matrix Σ, the values of which (σ^2) are inversely related to the integral under the visual acuity function around each letter. The posterior is then computed by comining the likelihood of the visual information $\mathcal{I}$ given a particular word, with a prior probability of that word $p(w)$ (e.g. derived from lexical frequency). This computation was performed using a log-odds formulation that exploits the proportionality in Bayes' rule to perform belief-updating without renormalisation (see Methods).

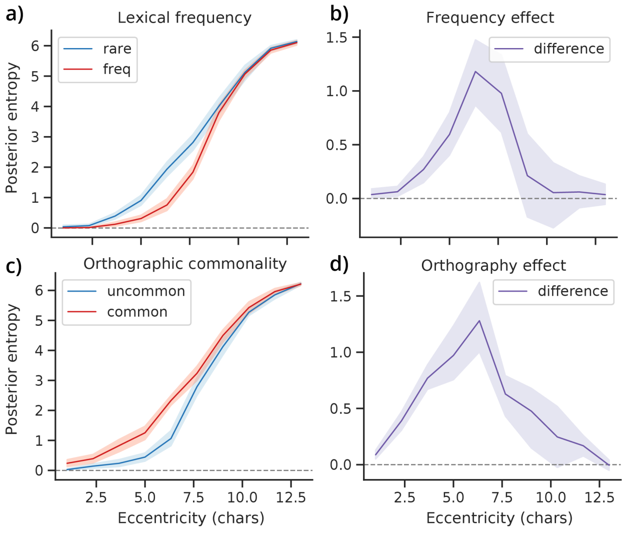

Figure A.3. Modulation of parafoveal identifiability by visual and linguistic features, and their interaction. The parafoveal entropy for a given word (Fig A.2) is a complex function that integrates linguistic and visual characteristics, and which can account for various known effects, such as the effect of lexical frequency and orthographic neighbourhood on visual word recognition. To illustrate this, we simulated some characteristic effects of eccentricity, frequency (**a,b**) and orthographic distinctiveness (**c,d**).
For frequency (**a**), we randomly sampled 20 'rare' and 'frequent' 5-letter words (based on a quartile split), and computed the parafoveal identifiability (quantified via posterior entropy) at increasing eccentricities. As can be seen, the percept becomes uncertain at increasing eccentricities more quickly for low-frequency words, showing that lexical frequency boosts parafoveal identifiability.
For orthography (**c**), we similarly sampled 20 7-letter words that were classified as orthographically common or uncommon based on the first three letters. Here, commonality was again defined using a quartile split but now on the number of alternative words starting with the same three letters. For instance, the letters 'awk' in the word 'awkward' are highly uncommon and allow to identify the entire word with high confidence based on just those three letters. As can be seen, the model predicts that orthographic uniqueness boosts parafoveal identifiability – as observed in experiments (see Schotter et al. (2012)).
Notably, when we consider the difference between the two classes of words (**b,d**), an inverted U shape is apparent: the effects are strongest at intermediate visibility. This demonstrates the well-established fact that the effects of prior (linguistic) knowledge is strongest at intermediate levels of perceptual uncertainty (see Norris (2006) for discussion). (Note that, while both the orthography and frequency effects are effects of "prior linguistic knowledge", only the frequency effect is technically an effect of the *prior*, since the orthography effect is driven by the generative model.) In all plots, thick lines represent the mean entropy across words; shaded regions indicate bootstrapped 95% confidence intervals.

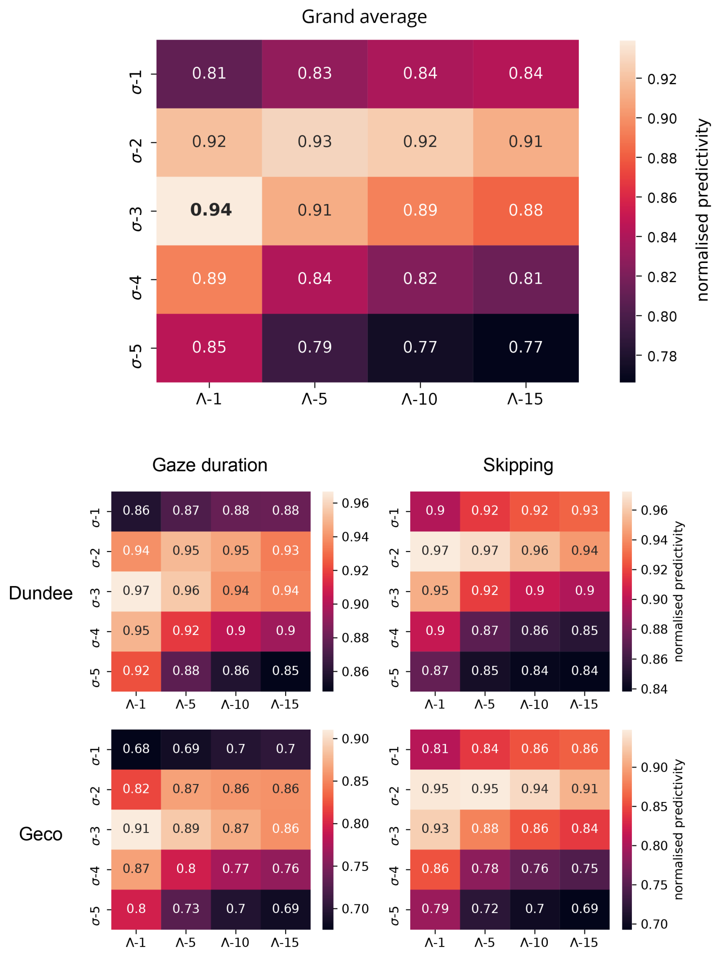

Figure A.4. Grid search to establish ideal observer parameters. Grid search result grand average (top) and individual results for different corpora and analyses (bottom). To decide on the values for σ and Λ, a grid search was performed on a random subset of 25% of the Dundee and Geco corpus; we did not apply it to PROVO because there was not enough data per participant. In both skipping and reading times, we performed a 10-fold cross-validation with the full model, using parafoveal entropy as computed with different visual acuity parameters σ and Λ (Equation 6). To avoid biasing the contextual vs non-contextual model comparison (Figure 6), we used both the contextual and non-contextual prior and averaged the results to obtain the results for each analysis in each corpus. To ensure that different analyses and corpora are weighted equally in the grand average, the prediction scores (R^2 or R^2_{McF}) were normalised by dividing the prediction score of each parameter combination by the highest score (i.e. score of the best parameter combination) for each subject, for each analysis. This resulted in $\sigma = 3$ and $\Lambda = 1$, which we have used in all analyses. Note that σ determines the perceptual span (see Figure A.2) and that $\sigma = 3$ corresponds well to what is known about the size of the perceptual span and is close to default parameters in other models (see Methods).

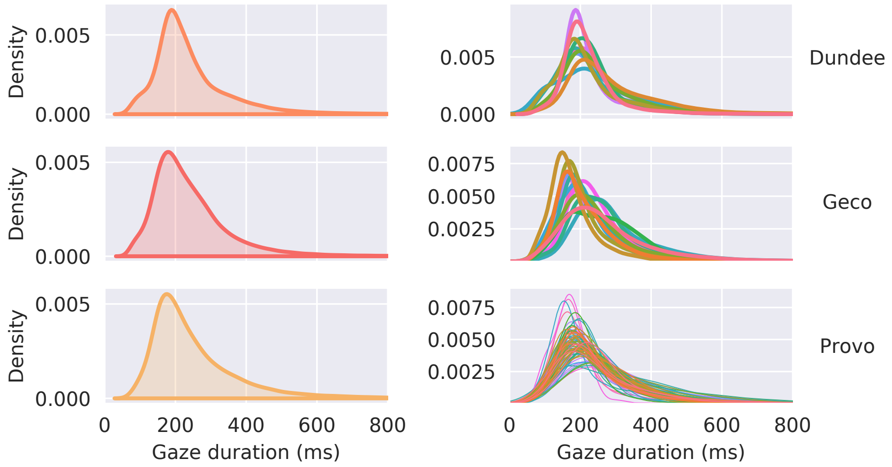

Figure A.5. Distributions of reading times (gaze durations). Kernel density estimate of the distribution of reading times across all datasets, both on average (left column) and in individual participants (right column).

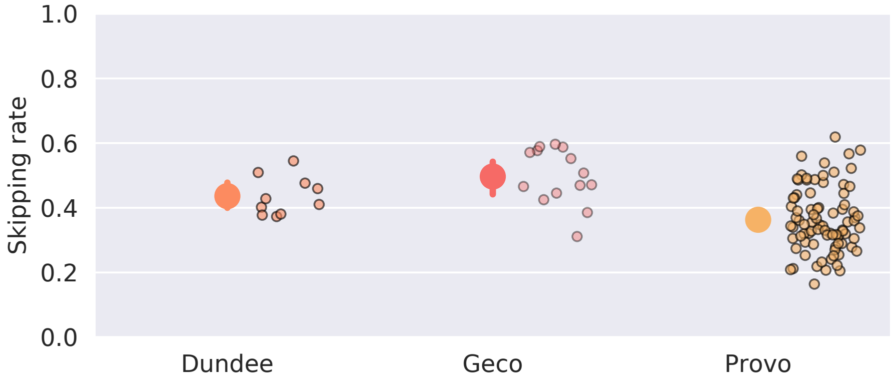

Figure A.6. Average skipping rate in each dataset. Average rate of skipping in all words included in the skipping analysis (see *Methods*) in all datasets. Large dots with error bar show group mean plus bootstrapped 95% confidence interval. Small dots show indididividual participants.

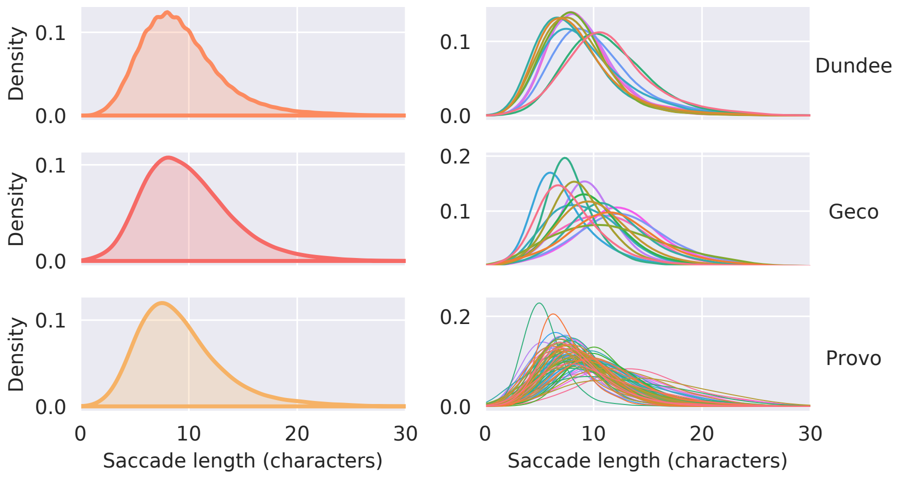

Figure A.7. Distribution of (forward) saccade lengths in each datasets Kernel density estimate of the distribution of saccade lengths (amplitudes) of first-pass, forward saccades in all datasets, both on average (left column) and per individual participant (right column). Note that for this visualisation we only included progressive, forward saccades within the same line (excluding saccades that cross lines), up to a maximum amplitude of 24 characters (excluding saccades during periods participants were not actually reading).

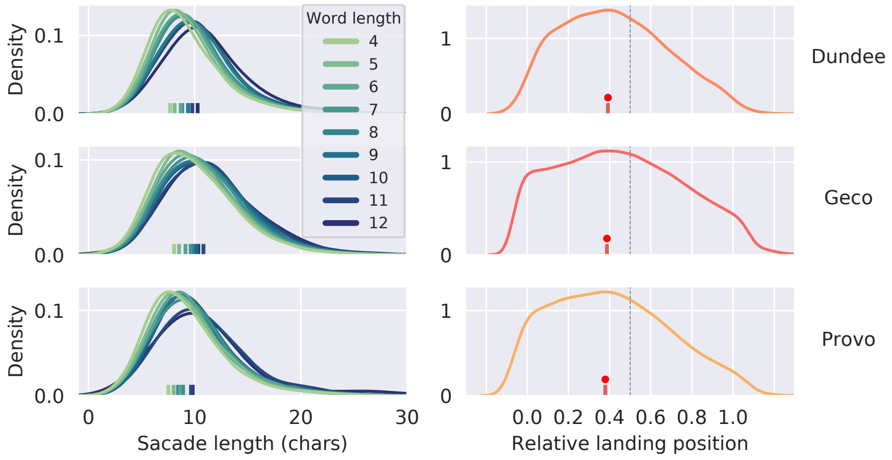

Figure A.8. Saccade lengths are tailored to word lengths and exhibit a preferred landing position.
Left column: Kernel density estimate of the saccade lengths, estimated separately for target words of different lengths. Colours indicate word lengths, vertical lines indicate the mode of the distribution. **Right column**: Kernel density estimate (plus mode) of the relative landing position, averaged across words with different lengths. Saccades are longer for longer words, such that a systematic 'preferred landing position' is maintained, slightly left to the center of the word (indicated by the vertical dashed line); see McConkie et al. (1988); Rayner (1979).

Model	Explanatory variable	Operationalisation
Prediction	Word predictability<br>Word predictability spillover (-1)<br>Word predictability spillover (-2)	Lexical surprisal<br>prior surprisal (-1)<br>prior surprisal (-2)
Preview	Prior parafoveal identifiability	Parafoveal entropy $H(p(w \mid preview))$
Baseline (non-contextual attributes of fixated word)	Log-lexical-frequency<br>Word log-frequency spillover (-1)<br>Word log-frequency spillover (-2)<br>Word class (function or content)<br>Word length<br>Absolute distance to OVP<br>Relative distance to OVP position	Unigram surprisal<br>Prior unigram surprisal (-1)<br>Prior unigram surprisal (-2)<br>Function (1) or content (1)<br>Word length (in characters)<br>Distance to mid-of-word in characters<br>Distance to mid-of-word in fraction

Table A.1. Explanatory variables for 3-way **reading times** analysis, comparing explanations for variation in reading times based on either two contextual sources of information about a word's identifiability: parafoveal preview or linguistic prediction, and based on non-contextual attributes of the fixated word.

Model	Explanatory variable	Operationalisation
Prediction	Lexical constraint	Prior lexical entropy
Preview	Prior parafoveal identifiability	Parafoveal entropy $H(p(w \mid preview))$
Baseline (occulomotor)	Word length<br>Word eccentricity	Word length (in characters)<br>Distance to prior fixation location

Table A.2. Explanatory variables for 3-way **skipping** analysis, contrasting explanations for skipping based a words prior identifiability based on parafoveal preview, a word's prior identifiability from constraint or contextual prediction, and low-level visual or oculomotor information. Note that, when we refer to 'full model' we simply mean the joint model combining all explanatory variables of the partial explanatory models.

Model	Explanatory variable	Operationalisation
Lexical processing ease	Lexical constraint<br>Prior parafoveal identifiability	Prior lexical entropy $H(p(w \mid context))$<br>Parafoveal entropy $H(p(w \mid preview))$
Oculomotor	Word length<br>Word eccentricity	Word length (in characters)<br>Distance to prior fixation location

Table A.3. Explanatory variables for 2-way **skipping** analysis, contrasting explanations for skipping based on factors determining a word's lexical processing ease (i.e. how well it can be predicted from context or discerned from a parafoveal preview) and explanations based on low-level visual or oculomotor information.

Model	Explanatory variable	Operationalisation
Lexical processing	Word predictability<br>Word frequency<br>Prior parafoveal identfiability<br>Word class	Lexical surprisal (+ spillovers)<br>Unigram surprisal (+ spillovers)<br>Prior parafoveal entropy<br>Function (0) or content (1)
Oculomotor	Absolute distance to OVP<br>Relative distance to OVP position<br>Word length*	Distance to mid-of-word in characters<br>Distance to mid-of-word in fraction<br>Word length (in characters)

Table A.4. Explanatory variables for 2-way **reading times** analysis, contrasting explanations for variation in reading times based on factors determining a word's lexical processing ease (e.g. frequency, or how well it can be predicted from context or discerned from a parafoveal preview) and low-level oculomotor factors. *Because length is an edge case, and reasonable arguments can be made either for or against inlcuding it in an oculomotor explanation for reading times, we ran two versions, one with (Fig 4) and one without (Fig A.12,A.13) length (see text)

.

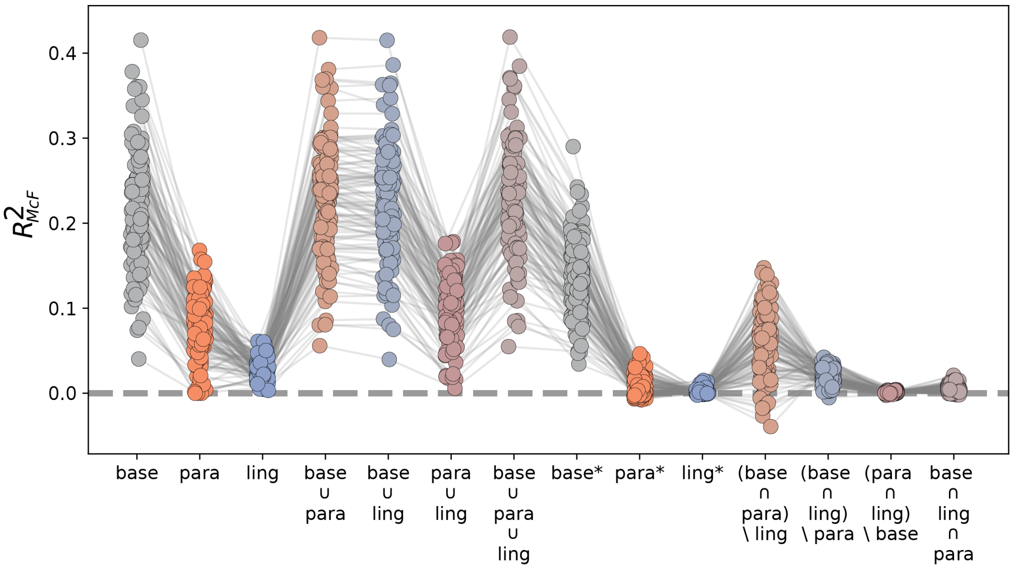

Figure A.9. Skipping variation partitioning for all participants. Explained cross-validated variation partition for skipping (see Fig 2) of each partition, for each participant, for the skipping analysis. Models for the baseline, parafoveal preview and linguistic prediction are indicated by 'base', 'para', and 'ling', respectively. Unions are indicated by ∪, intersections by ∩; for the relative complement we use the asterisk-notation: e.g. 'para*' indicates variation explained uniquely by parafoveal preview. Note that due to cross-validation, the amount of variation explained can become negative in some partitions for individual participants (see Methods).

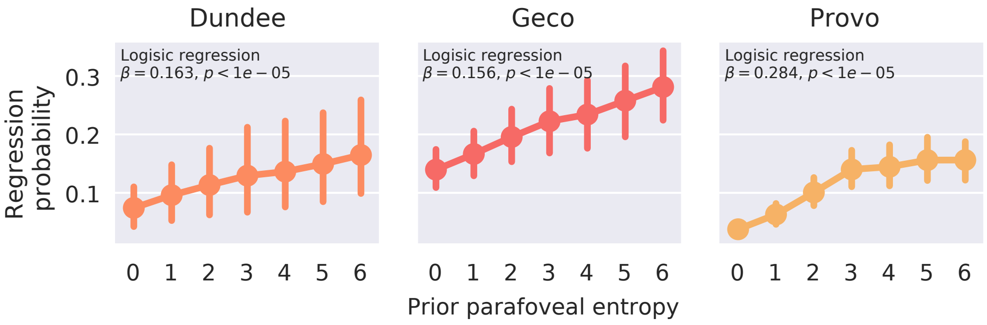

Figure A.10. Probability that a skipped word is regressed to depends on its prior identifiability.
Probability that an initially skipped word is subsequently fixated (i.e. regressed to), as a function of the prior parafoveal entropy, before skipping. Dots with connecting lines show the average regression probability for initially skipped words as a function of the binned prior parafoveal entropy. Error bars show the (bootstrapped) 95% confidence interval around the mean (across participants). In all datasets, the probability that a skipped word gets subsequently fixated depends on the amount of visual information about word identity that was available *before* the word was was skipped, suggesting a compensation mechanism. Note that the binning is done for visualisation purposes only. Statistical evaluation is based on a subject-wise logistic regression on the word-by-word parafoveal entropy and regression values. Statistical significance is established by a bootstrap test on the subjects' coefficients, in each dataset.

Table A.5: Literature sample for effect size ranges

**Effect type**	**Publication**	**Effect size**
preview benefit	Inhoff, A. W. (1989). Lexical access during eye fixations in reading: Are word access codes used to integrate lexical information across interword fixations?. Journal of Memory and Language, 28(4), 444-461.	51
preview benefit	Veldre, A., & Andrews, S. (2018). Parafoveal preview effects depend on both preview plausibility and target predictability. Lexical access during eye fixations in reading: Quarterly Journal of Experimental Psychology, 71(1), 64-74.	49
preview benefit	Inhoff, A. W., & Rayner, K. (1986). Parafoveal word processing during eye fixations in reading: Effects of word frequency. Perception & psychophysics, 40(6), 431-439.	40
preview benefit	McDonald, S. A. (2006). Parafoveal preview benefit in reading is only obtained from the saccade goal. Vision Research, 46(26), 4416-4424.	35

Continued on next page

Table A.5 – *Continued from previous page*

Effect type	Publication	Effect size
preview benefit	Williams, C. C., Perea, M., Pollatsek, A., & Rayner, K. (2006). Previewing the neighborhood: The role of orthographic neighbors as parafoveal previews in reading. Journal of Experimental Psychology: Human Perception and Performance, 32(4), 1072.	26.7
preview benefit	Kennison, S. M., & Clifton, C. (1995). Determinants of parafoveal preview benefit in high and low working memory capacity readers: Implications for eye movement control. Journal of Experimental Psychology: Learning, Memory, and Cognition, 21(1), 68.	25.25
preview benefit	Blanchard, Harry E., Alexander Pollatsek, and Keith Rayner. "The acquisition of parafoveal word information in reading." Perception & Psychophysics 46.1 (1989): 85-94.	22.6
preview benefit	Schroyens, W., Vitu, F., Brysbaert, M., & d'Ydewalle, G. (1999). Eye movement control during reading: Foveal load and parafoveal processing. The Quarterly Journal of Experimental Psychology Section A, 52(4), 1021-1046.	14.6
prediction benefit	Ehrlich, S. F., & Rayner, K. (1981). Contextual effects on word perception and eye movements during reading. Journal of verbal learning and verbal behavior, 20(6), 641-655.	33
prediction benefit	Rayner, K., & Well, A. D. (1996). Effects of contextual constraint on eye movements in reading: A further examination. Psychonomic Bulletin & Review, 3(4), 504-509.	20
prediction benefit	RJ. Altarriba, J. Kroll, A. Sholl, K. Rayner. (1996) The influence of lexical and conceptual constraints on reading mixed-language sentences: Evidence from eye fixations and naming times Memory & Cognition, 24 (1996), pp. 477-492.	21
prediction benefit	Ashby, J., Rayner, K., & Clifton Jr, C. (2005). Eye movements of highly skilled and average readers: Differential effects of frequency and predictability. The Quarterly Journal of Experimental Psychology Section A, 58(6), 1065-1086.	23.5
prediction benefit	Rayner, K., Ashby, J., Pollatsek, A., & Reichle, E. D. (2004). The effects of frequency and predictability on eye fixations in reading: implications for the EZ Reader model. Journal of Experimental Psychology: Human Perception and Performance, 30(4), 72	19

Continued on next page

Table A.5 – *Continued from previous page*

**Effect type**	**Publication**	**Effect size**
prediction benefit	Rayner, K., Binder, K. S., Ashby, J., & Pollatsek, A. (2001). Eye movement control in reading: Word predictability has little influence on initial landing positions in words. Vision Research, 41(7), 943-954.	15
prediction benefit	Rayner, K., Slattery, T. J., Drieghe, D., & Liversedge, S. P. (2011). Eye movements and word skipping during reading: effects of word length and predictability. Vision Research, 41(7), 943-954.	18
prediction benefit	Hand, C. J., Miellet, S., O'Donnell, P. J., & Sereno, S. C. (2010). The frequency-predictability interaction in reading: It depends where you're coming from. Journal of Experimental Psychology: Human Perception and Performance, 36(5), 12941313.	12

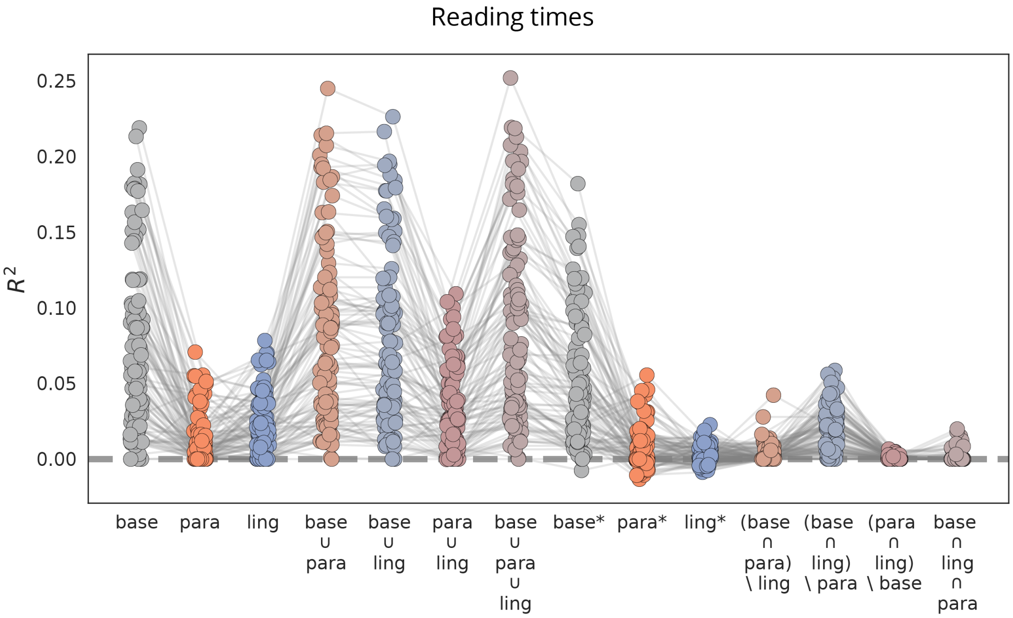

Figure A.11. Reading times variance partitioning. Explained cross-validated variation partition for skipping (see Fig 3) of each partition, for each participant, for the skipping analysis. Models for the baseline, parafoveal preview and linguistic prediction are indicated by 'base', 'para', and 'ling', respectively. Unions are indicated by ∪, intersections by ∩; for the relative complement we use the asterisk-notation: e.g. 'para*' indicates variation explained uniquely by parafoveal preview (see Methods). Note that due to cross-validation, the amount of variation explained can become negative in individual participants (see Methods).

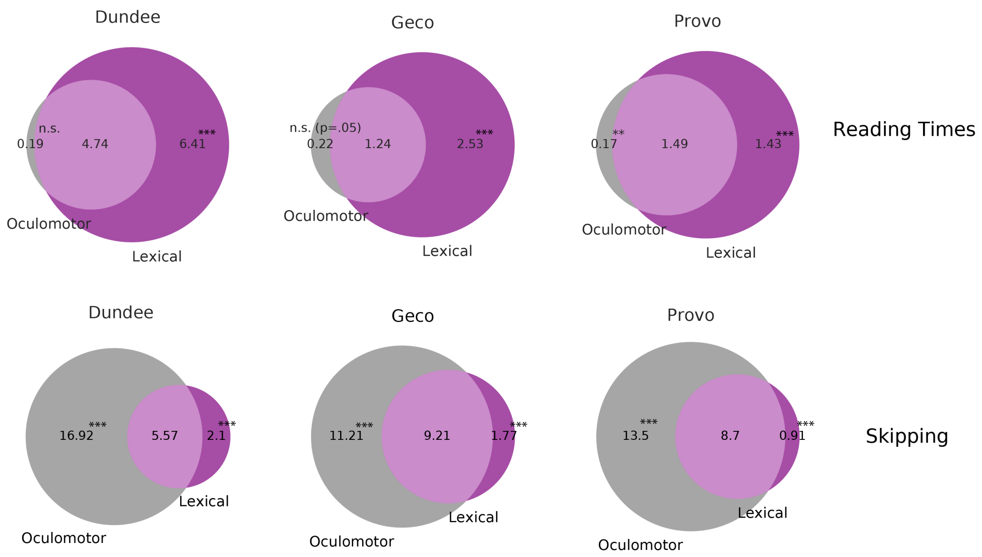

Figure A.12. Two-way partition of reading times and skipping on individual datasets. Upper row: unique explained variation for the oculomotor model (Dundee: 0.19% bootstrap 95CI: 0.0 – 0.55%; bootstrap t-test: $p < 10^{-5}$; Geco: 0.22%, 95CI: 0.0 – 0.59%; $p = 0.051$; Provo: 0.17%; 95CI: 0.12 – 0.55%, $p = 0.0012$). Upper row: unique explained variation for the lexical model (Dundee: 6.41% 95CI: 4.7 – 8.1%; p=0.07; Geco: 2.53%, 95CI: 1.86 – 3.16%; $p < 10^{-5}$; Provo: 2.67%; 95CI: 2.03 – 3.37%,$p < 10^{-5}$). Lower row: unique explained skipping variation for the oculomotor model (Dundee: 16.92% bootstrap 95CI: 14.86 – 18.93%, bootstrap t-test compared to zero: $p < 10^{-5}$; Geco: 11.21%, 95CI: 10.26 – 12.19%, $p < 10^{-5}$; Provo: 13.50%; 95CI: 12.47 – 14.55%, $p < 10^{-5}$). Lower row: unique explained skipping variation for the lexical model (Dundee: 2.10% 95CI: 1.64 – 2.54%, $p < 10^{-5}$; Geco: 1.77%, 95CI: 1.25 – 2.34%; $p < 10^{-5}$; Provo: 0.91%; 95CI: 0.69 – 1.13%, $p < 10^{-5}$).

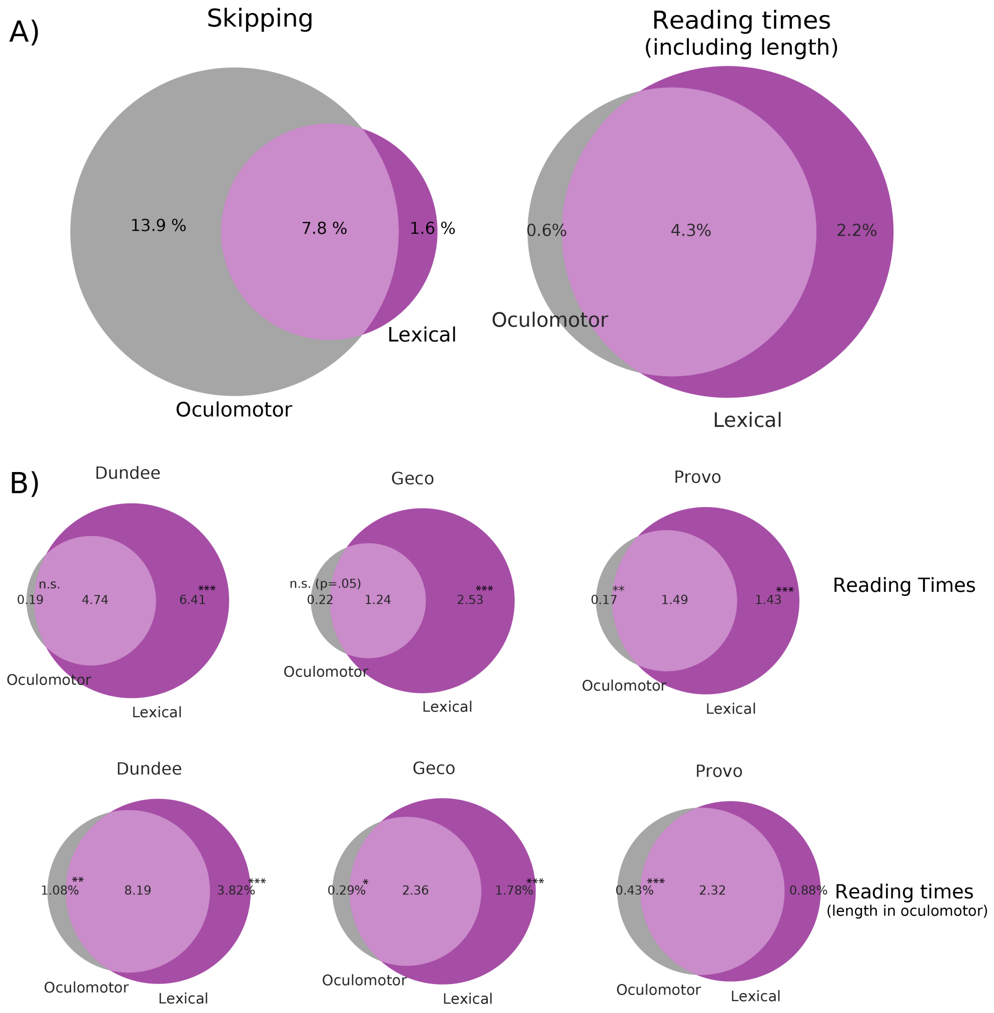

Figure A.13. Two-way partition with and without length in the oculomotor model of reading times. Including word length in the oculomotor model for reading times leads to an increase in variance explained, most of which is overlapping with the lexical model, due to the correlation with frequency. However, the overall dissociation remains (see text). (**a**)) Grand average across datasets. Same as Figure 4, but including word length in the oculomotor model of reading times. (**b**)) Individual datasets, with (lower row) and without (upper row) including word length as an explanatory variable in the oculomotor model. Upper row: unique explained variation for the oculomotor model (Dundee: 0.19% bootstrap 95CI: $0.0 - 0.55\%$; bootstrap t-test: $p < 10^{-5}$; Geco: 0.22%, 95CI: $0.0 - 0.59\%$; $p = 0.051$; Provo: 0.17%; 95CI: $0.12 - 0.55\%$, $p = 0.0012$). Upper row: unique explained variation for the lexical model (Dundee: 6.41% 95CI: $4.7 - 8.1\%$; p=0.07; Geco: 2.53%, 95CI: $1.86 - 3.16\%$; $p < 10^{-5}$; Provo: 2.67%; 95CI: $2.03 - 3.37\%$,$p < 10^{-5}$). Lower row: unique explained variation for the oculomotor model (Dundee: 1.08% 95CI: $0.24 - 2.214\%$; $p < 10^{-5}$; Geco: 0.29%, 95CI: $0.009 - 0.67\%$; $p = 0.015$; Provo: 0.78%; 95CI: $0.41 - 1.20\%$, $p < 10^{-5}$). Lower row: unique explained variation for the lexical model (Dundee: 3.82% 95CI: $2.64 - 5.56\%$; $p < 10^{-5}$; Geco: 1.78%, 95CI: $1.31 - 2.25\%$; $p < 10^{-5}$; Provo: 1.57%; 95CI: $1.06 - 2.08\%$,$p < 10^{-5}$).
